# Supplementary material for: TRIB3 promotes MYC-associated lymphoma development through suppression of UBE3B-mediated MYC degradation
Source: Nat Commun. 2020 Dec 9;11:6316. doi: 10.1038/s41467-020-20107-1 (PMC7725785; doi:10.1038/s41467-020-20107-1)
Supplement: Supplementary file 1 — Supplementary Informaiton [file 41467_2020_20107_MOESM1_ESM.pdf]

Supplementary Figures and Legends

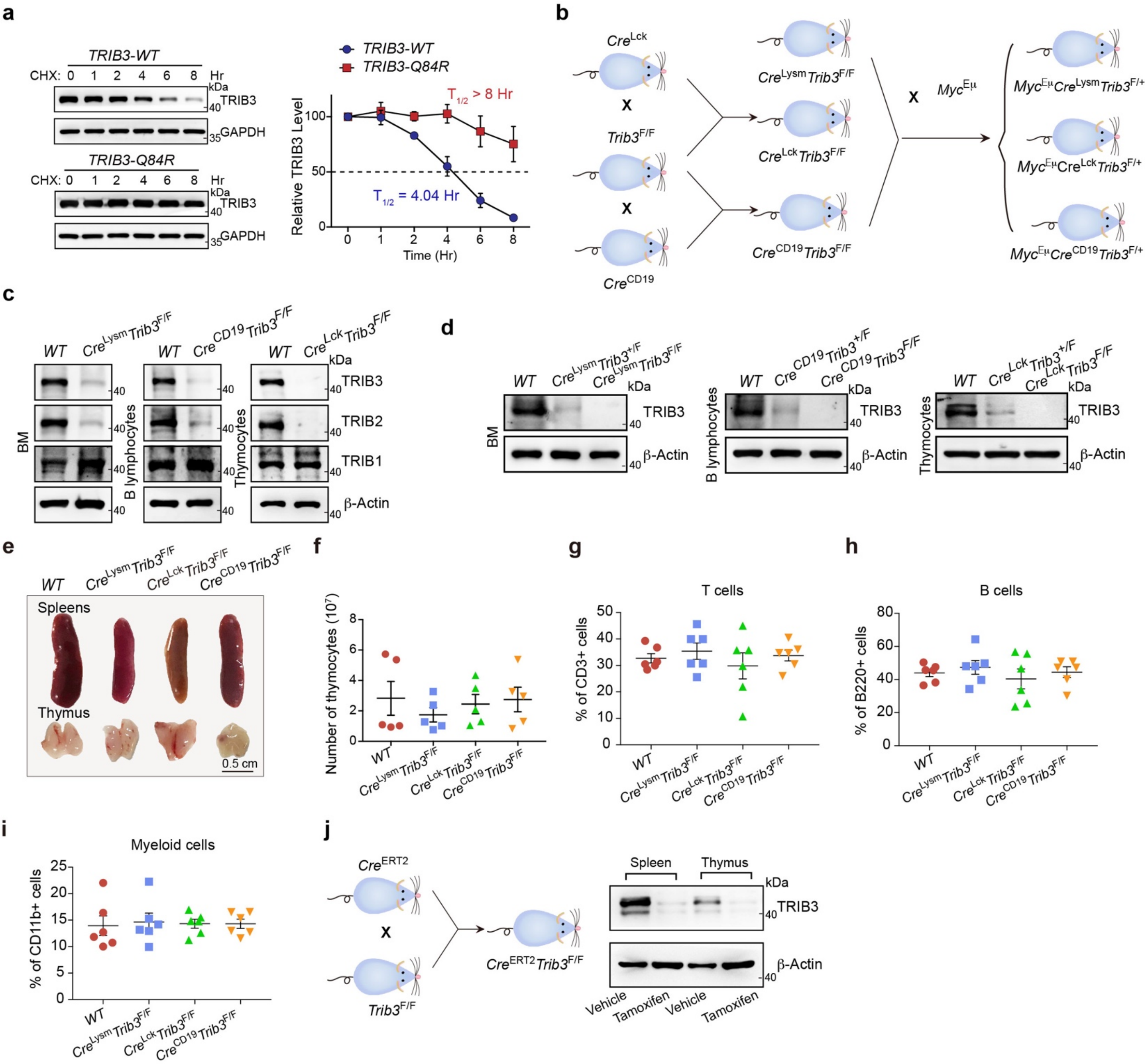

Supplementary Figure 1. Loss of Trib3 in Different Cell Lineages. Related to Fig. 1. (**a**) Q84R mutation increased the stability of TRIB3 protein. HEK 293T cells were transfected with the indicated plasmids, and 12 hr later cells were incubated with CHX (10 μg/mL) for the indicated times. The data are presented as the means ±S.E.M from 3 independent experiments. (**b**) Schematic strategy for generation of myeloid cell-specific deletion of *Trib3* (*Cre*^{lysm}*Trib3*$^{\mathrm{F/F}}$), thymocyte-specific deletion of *Trib3* (*Cre*^{Lck}*Trib3*$^{\mathrm{F/F}}$), or B lymphocyte-specific deletion of *Trib3* (*Cre*$^{\mathrm{CD19}}$*Trib3*$^{\mathrm{F/F}}$). (**c**) TRIB3, TRIB2 and TRIB1 expression levels in BM, B lymphocytes, and thymocytes from the indicated mice. The data are presented as representative from 3 independent experiments. (**d**) TRIB3

expression in BM, B lymphocytes, and thymocytes from the indicated mice. The data are presented as representative from 3 independent experiments. (**e**) Gross appearance of the spleens and thymuses of the indicated mice at 5 weeks of age. Scale bar, 0.5 cm. (**f**) Total number of thymocytes for the individual indicated mice at 5 weeks of age (n = 5 per group). Data are represented as means ± SEM. (**g-i**) Spleens from 5-week old indicated mice (n = 6 per group). were digested, homogenised and stained with anti-CD3 APC, anti-B220 FITC, and anti-CD11b PE antibodies to detect T (**g**), B (**h**), and myeloid (**i**) cell subsets, respectively. The percentage of each cell population was determined via flow cytometry analysis. Data are represented as means ± SEM. (**j left**) Schematic strategy for generation of $Cre^{ERT2}Trib3^{F/F}$ mice (n = 6 per group). (**j right**) TRIB3 expression levels in spleens and thymuses from $Myc^{E\mu}Cre^{ERT2}Trib3^{F/+}$ mice (6 months old) with or without tamoxifen. The data are presented as representative from 3 independent experiments.

Source data are provided as a Source Data file.

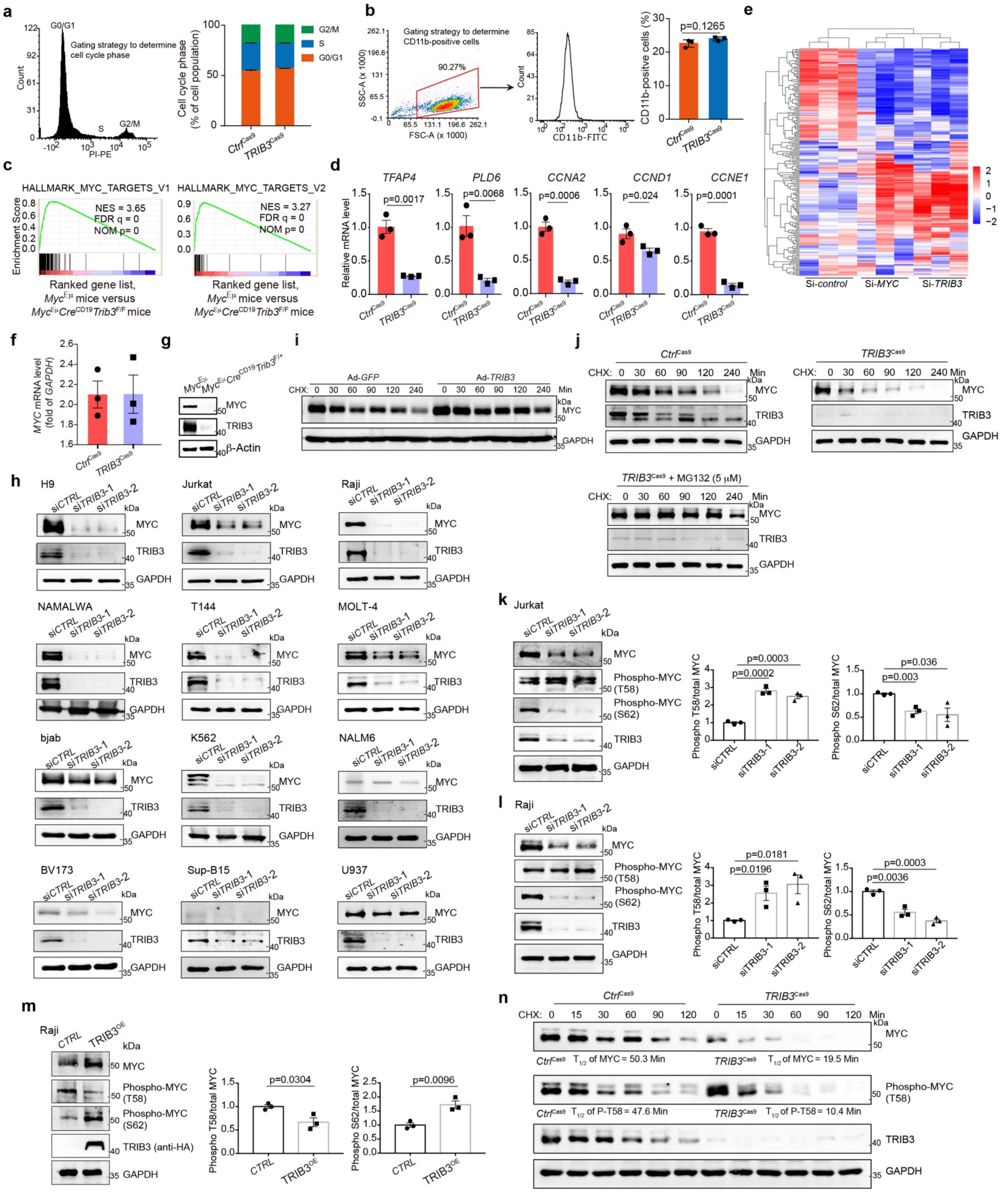

Supplementary Figure 2. TRIB3 Supports the MYC Stability. Related to Fig. 2 and 3. (a) Flow cytometric analysis of DNA content for cell-cycle progression from propidium iodide (PI, 100 μg/ml) staining of Raji cells

with or without *TRIB3* deletion. Data are means ± S.E.M of 3 independent experiments. **(b)** Flow cytometric analysis of cell differentiation from CD11b staining of Raji cells with or without *TRIB3* deletion. Data are means ± S.E.M of 3 independent experiments. Statistical significance was determined by two-tailed Student's t test. **(c)** GSEA shows global downregulation of MYC target genes in the $Myc^{E\mu}Cre^{CD19}Trib3^{F/F}$ vs. $Myc^{E\mu}$ groups. **(d)** The mRNA levels of *MYC* target genes in the indicated cells with or without *TRIB3* deletion. The data are presented as the means ±S.E.M from 3 independent experiments. Statistical significance was determined by two-tailed Student's t test. *P* values were indicated in this panel. **(e)** Clustering and heatmap of MYC target genes (HALLMARK MYC TARGETS) in the indicated cells. **(f)** *MYC* mRNA levels in the indicated cells with or without *TRIB3* deletion. The data are presented as the mean ±S.E.M from 3 independent experiments. **(g)** MYC protein levels in the indicated cells with or without *Trib3* deletion. The data are presented as presentative from 3 independent experiments. **(h)** MYC protein levels in the indicated cells with or without *TRIB3* depletion. 11 lymphoma and leukemia cells and primary lymphoma cells (T144) were transfected with *control* siRNA or *TRIB3* siRNA1/2 for 24 hr, and the MYC protein level was detected by the immunoblotting assay. The data are presented as representative from 3 independent experiments. **(i)** Representative results of MYC degradation in Raji cells with or without *TRIB3* overexpression analyzed by Western blotting from 3 independent experiments. **(j)** Representative results of MYC degradation in Raji cells with or without *TRIB3* deletion analyzed by Western blotting from 3 independent experiments. **(k and l)** Western blot analysis of MYC S62 and T58 phosphorylation and total MYC levels in Jurkat **(k)** or Raji **(l)** cells 72 hr after transfected with scrambled or TRIB3 siRNA1/2. Levels were quantitated using ImagePro Plus (IPP) 6.0 software, and ratio of S62/total MYC or T58/ total MYC was calculated. Shown is means ± S.E.M of 3 independent experiments. Statistical significance was determined by two-tailed Student's t test. *P* values were indicated in panels **k** and **l**. **(m)** Western blot analysis of MYC S62 and T58 phosphorylation and total MYC levels in Raji cells stably overexpressing TRIB3. Levels were quantitated using ImagePro Plus (IPP) 6.0 software, and ratio of S62/total MYC or T58/ total MYC was calculated. Shown is means ± S.E.M of 3 independent experiments. Statistical significance was determined by two-tailed Student's t test. *P* values were indicated in this panel. **(n)** Effect of *TRIB3* deletion on MYC and p-T58 MYC degradation *in vivo*. *Control* or *TRIB3*-deleted Raji cells were incubated with CHX (10 μg/mL) for the indicated times. The MYC and p-T58 MYC proteins were detected by Western blot; GAPDH was used as a loading control. The data are presented as the means ±S.E.M from 3 independent experiments.

Source data are provided as a Source Data file.

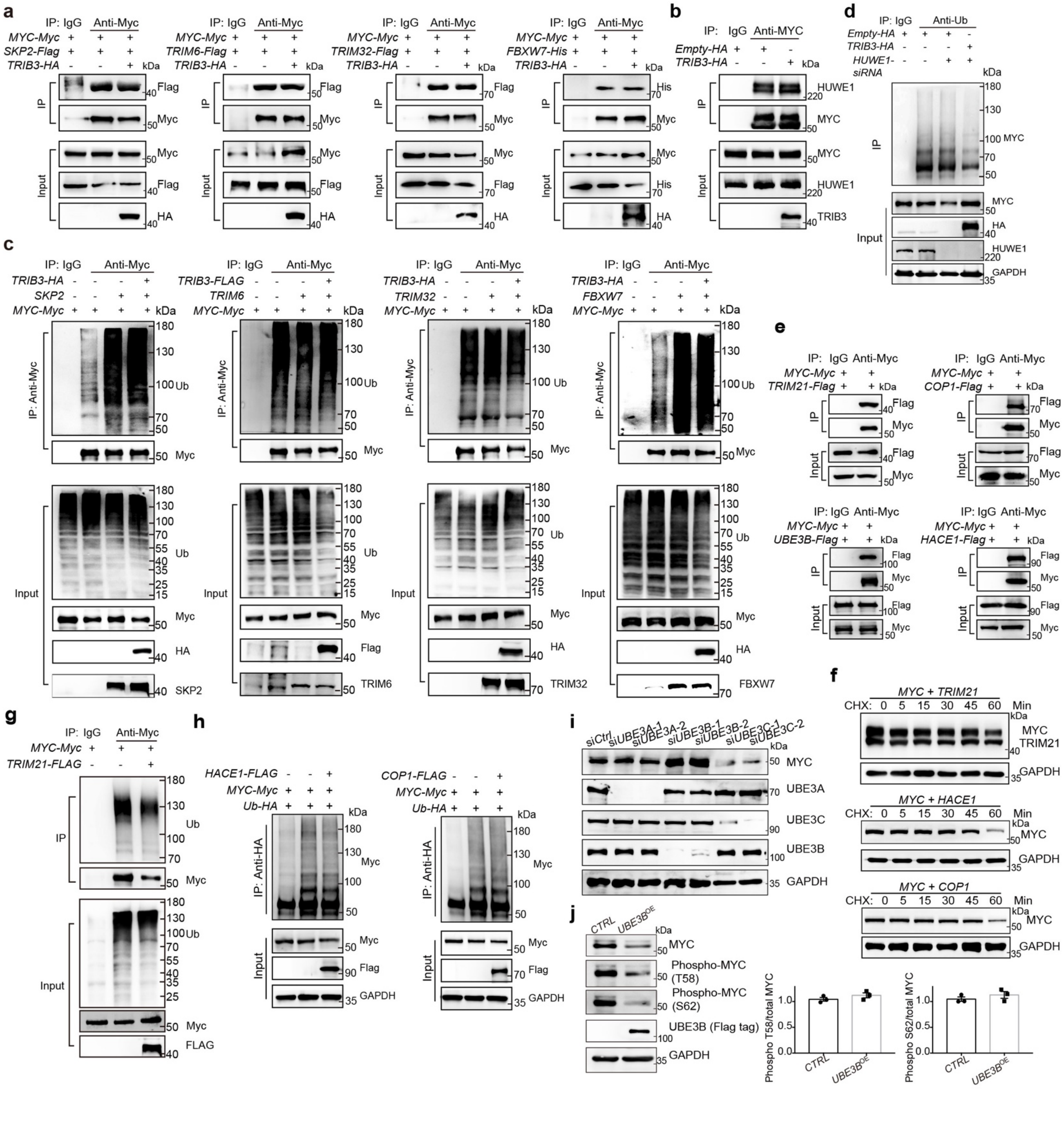

Supplementary Figure 3. TRIB3 Shows No Effects on the Axis of MYC and Known E3 Ligases. Related to Fig. 3. (**a**) The effects of TRIB3 overexpression on the interaction of MYC and the indicated E3 ligases. HEK 293T cells were transfected with the indicated plasmids, and the interaction of MYC and known E3 ligases was detected by Co-IP assays. (**b**) The effects of TRIB3 overexpression on the interaction of MYC and HUWE1 E3 ligase. Raji cells were transfected with the indicated plasmids, and the interaction of MYC and HUWE1 was

detected by Co-IP assays. (**c**) The effects of TRIB3 overexpression on MYC ubiquitination mediated by the indicated E3 ligases. (**d**) The effects of TRIB3 overexpression on MYC ubiquitination of *HUWE1* depleted cells. Raji cells were transfected with the indicated siRNAs, and the MYC ubiquitination was detected by Co-IP assays. (**e**) The interaction of MYC and TRIM21, UBE3B, COP1, or HACE1 was detected by Co-IP assays. HEK 293T cells were transfected with the indicated plasmids, and the interactions was detected by Co-IP assays. (**f**) The effect of TRIM21, HACE1, or COP1 overexpression on MYC degradation at the indicated times. (**g, h**) The effects of TRIM21, HACE1, or COP1 overexpression on the ubiquitination of MYC. HEK 293T cells were transfected with the indicated plasmids, and the ubiquitination of MYC was detected by Co-IP assays. (**i**) The effects of *UBE3A*, *UBE3C, UBE3B* depletion on MYC protein level in the Raji cells. **(j)** Western blot analysis of MYC S62 and T58 phosphorylation and total MYC levels in Raji overexpressing UBE3B. Levels were quantitated using ImagePro Plus (IPP) 6.0 software, and ratio of S62/total MYC or T58/ total MYC was calculated. For panels **a-j**, shown is representative and/or means ± S.E.M of 3 independent experiments.

Source data are provided as a Source Data file.

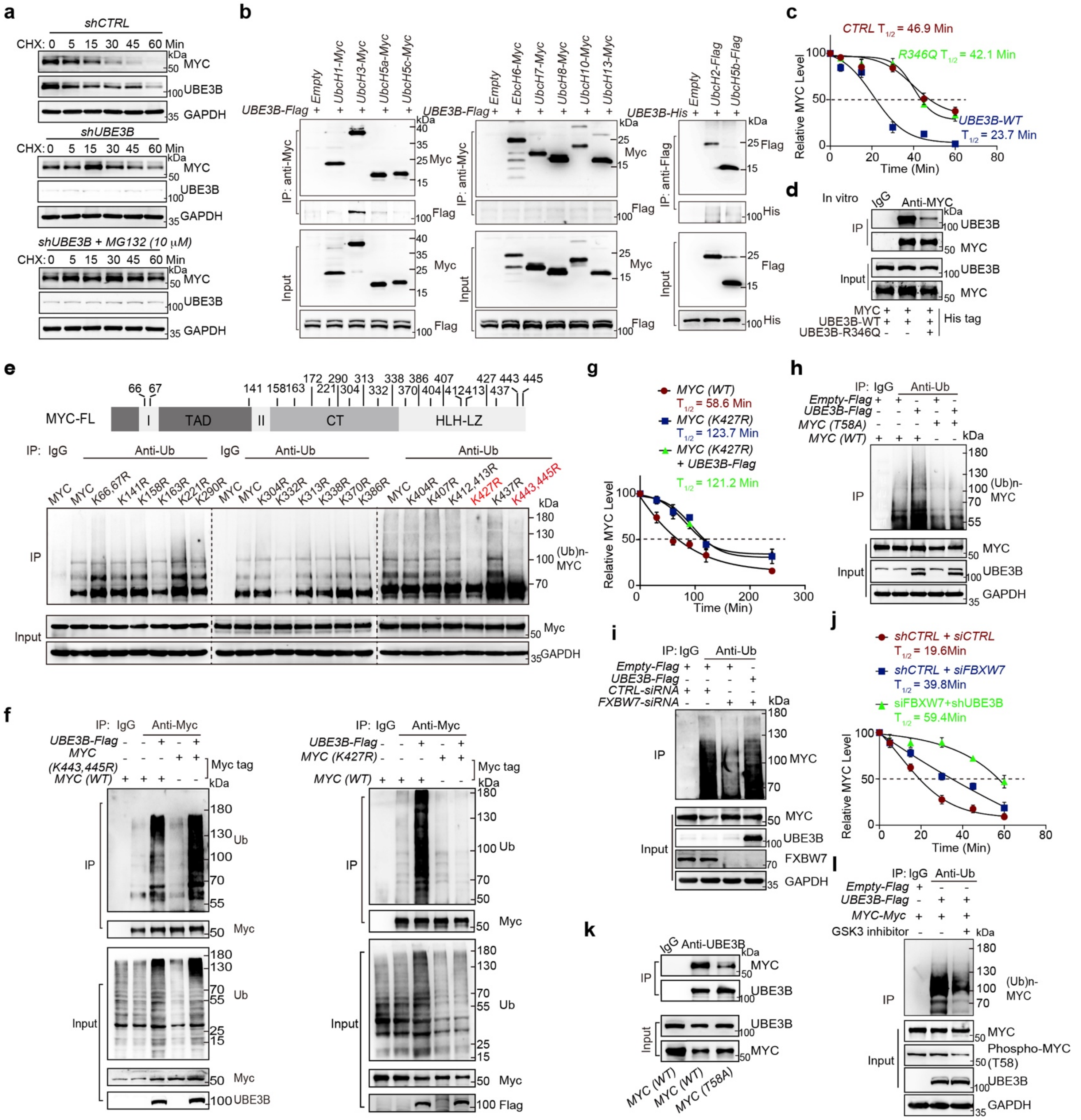
a
shCTRL
CHX: 0 5 15 30 45 60 Min
kDa
MYC
UBE3B
GAPDH
shUBE3B
shUBE3B + MG132 (10 μM)
b
UBE3B-Flag
Empty
UbcH1-Myc
UbcH3-Myc
UbcH5a-Myc
UbcH5c-Myc
EbcH6-Myc
UbcH7-Myc
UbcH8-Myc
UbcH10-Myc
UbcH13-Myc
UBE3B-His
UbcH2-Flag
UbcH5b-Flag
IP: anti-Myc
IP: anti-Flag
Input
Myc
Flag
His
c
CTRL T1/2 = 46.9 Min
R346Q T1/2 = 42.1 Min
UBE3B-WT T1/2 = 23.7 Min
Relative MYC Level
Time (Min)
d
In vitro
IgG
Anti-MYC
IP
Input
UBE3B
MYC
UBE3B-WT
UBE3B-R346Q
His tag
e
MYC-FL
I
TAD
II
CT
HLH-LZ
66 67 141 158 163 172 221 290 304 313 332 338 370 386 404 407 412 413 427 437 443 445
IP: IgG Anti-Ub
MYC K66,67R K141R K158R K163R K221R K290R K304R K332R K313R K338R K370R K386R K404R K407R K412,413R K427R K437R K443,445R
(Ub)n-MYC
Input
Myc
GAPDH
f
IP: IgG Anti-Myc
UBE3B-Flag
MYC (K443,445R)
MYC (WT)
MYC (K427R)
Myc tag
Ub
g
MYC (WT) T1/2 = 58.6 Min
MYC (K427R) T1/2 = 123.7 Min
MYC (K427R) + UBE3B-Flag T1/2 = 121.2 Min
h
IP: IgG Anti-Ub
Empty-Flag
UBE3B-Flag
MYC (T58A)
MYC (WT)
(Ub)n-MYC
i
CTRL-siRNA
FXBW7-siRNA
FXBW7
j
shCTRL + siCTRL T1/2 = 19.6Min
shCTRL + siFBXW7 T1/2 = 39.8Min
siFBXW7+shUBE3B T1/2 = 59.4Min
k
Anti-UBE3B
MYC (WT)
MYC (T58A)
l
MYC-Myc
GSK3 inhibitor
Phospho-MYC (T58)

Supplementary Figure 4. UBE3B Induced MYC Ubiquitination at Lysine 427. Related to Fig. 3. (**a**) *UBE3B* depletion increased the MYC stability. *Control* or *UBE3B*-depleted Raji cells were incubated with CHX (10 μg/mL) or CHX plus MG132 (10 μM) for the indicated times. The MYC protein was detected by Western blot analysis with GAPDH as a loading control. (**b**) Co-immunoprecipitation assays between UBE3B and 11 E2 ubiquitin-conjugating enzymes show that UBCH3 but not other E2 enzymes has the binding ability to UBE3B. (**c**) UBE3B R346Q mutation did not degraded MYC protein effectively compared the wildtype of MYC. (**d**) The direct interaction of UBE3B and MYC in the purified system. (**e**) Identifying the ubiquitinated sites of MYC protein. HEK 293T cells were transfected with the indicated mutants of MYC, and the ubiquitination of MYC was detected by Co-IP assays. (**f**) The effects of UBE3B on the ubiquitination of MYC K443/445R and K427R mutants. HEK 293T cells were transfected with the indicated mutants of MYC, and the ubiquitination of MYC was detected by Co-IP assays. (**g**) The effects of UBE3B on the degradation of MYC K427R mutant. HEK 293T cells were transfected with the indicated mutants of MYC, and the degradation of MYC was detected by western blotting. (**h**) UBE3B-mediated MYC ubiquitination depended on the MYC phosphorylation at T58. (**i**) The effects of UBE3B on the ubiquitination of MYC in *FBXW7*-depleted cells. Raji cells were transfected with the indicated siRNAs and plasmids, and the ubiquitination of MYC was detected by Co-IP assays. (**j**) The effects of UBE3B on the degradation of MYC in *FBXW7*-depleted cells. Raji cells with indicated depletion were incubated with CHX (10 μg/mL) or CHX plus MG132 (10 μM) for the indicated times. The MYC protein was detected by Western blot analysis with GAPDH as a loading control. (**k**) T58A mutation decreased the interaction of MYC and UBE3B. HEK 293T cells were transfected with the indicated mutants of MYC (Myc tagged) and UBE3B (Flag tagged), and the interaction of MYC and UBE3B was detected by Co-IP assays. (**l**) GSK3 inhibitor decreased UBE3B-mediated MYC ubiquitination. HEK 293T cells were transfected indicated plasmids for 12 hr, and then incubated with GSK3 inhibitor (CT99021, 10 μM) for 12 hr. The MYC ubiquitination was detected by Co-IP assays. For panels **a-l**, shown is representative and/or means ± S.E.M of 3 independent experiments.

Source data are provided as a Source Data file.

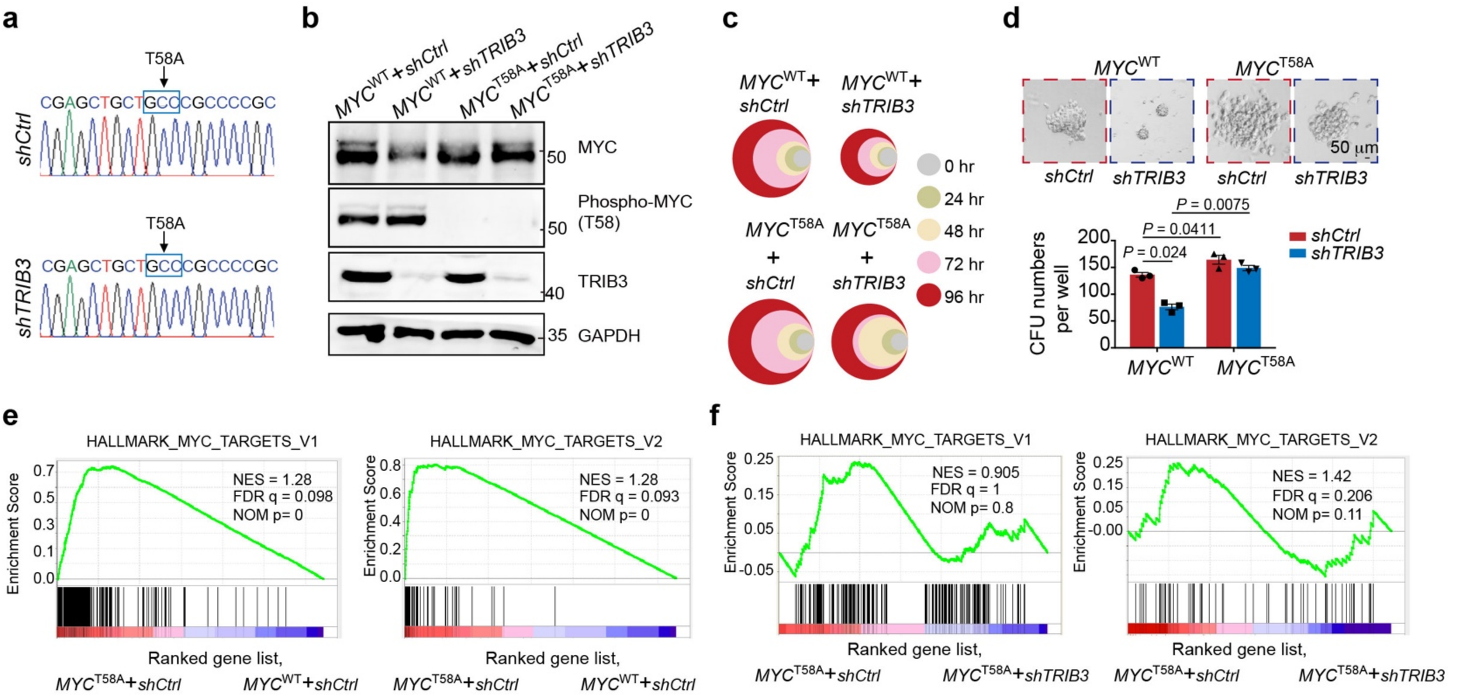

Supplementary Figure 5. T58A Mutation Alleviated the Dependence of MYC Pathway-related Gene Expression on TRIB3. Related to Fig. 3. (**a**) Sequencing verification of the codon replacement by CRISPR-Cas9 resulting in MYC (T58A). (**b**) Western blot analysis of MYC T58 phosphorylation and total MYC levels in *MYC*^{WT}*+shCTRL, MYC*^{WT}*+shTRIB3*, *MYC*^{T58A}*+shCTRL,* and *MYC*^{T58A}*+shTRIB3* Raji cells. Shown is representative of 3 independent experiments. (**c**) Relative cell viabilities of *MYC*^{WT}*+shCTRL, MYC*^{WT}*+shTRIB3*, *MYC*^{T58A}*+shCTRL,* and *MYC*^{T58A}*+shTRIB3* Raji cells for the indicated times. The colors represent different time points; the diameter indicates the relative cell viability. (**d**) Representative images and colony numbers of LCs from the indicated groups with or without *TRIB3* deletion. Scale bar, 50 μm. Shown is means ± S.E.M of 3 independent experiments. Statistical significance was determined by two-tailed Student's t test. *P* value: 0.024 (*MYC*^{WT}*+shCTRL vs. MYC*^{WT}*+shTRIB3*), 0.0411 (*MYC*^{WT}*+shCTRL vs. MYC*^{T58A}*+shCTRL),* 0.0075 (*MYC*^{WT}*+shTRIB3 vs. MYC*^{T58A}*+shTRIB3*). (**e**) GSEA shows global upregulation of MYC target genes in *MYC*^{T58A}*+shCTRL* Raji cells versus *MYC*^{WT}*+shCTRL* Raji cells. (**f**) GSEA shows no difference of MYC target genes in *MYC*^{T58A}*+shCTRL* Raji cells versus *MYC*^{T58A}*+shTRIB3* Raji cells.

Source data are provided as a Source Data file.

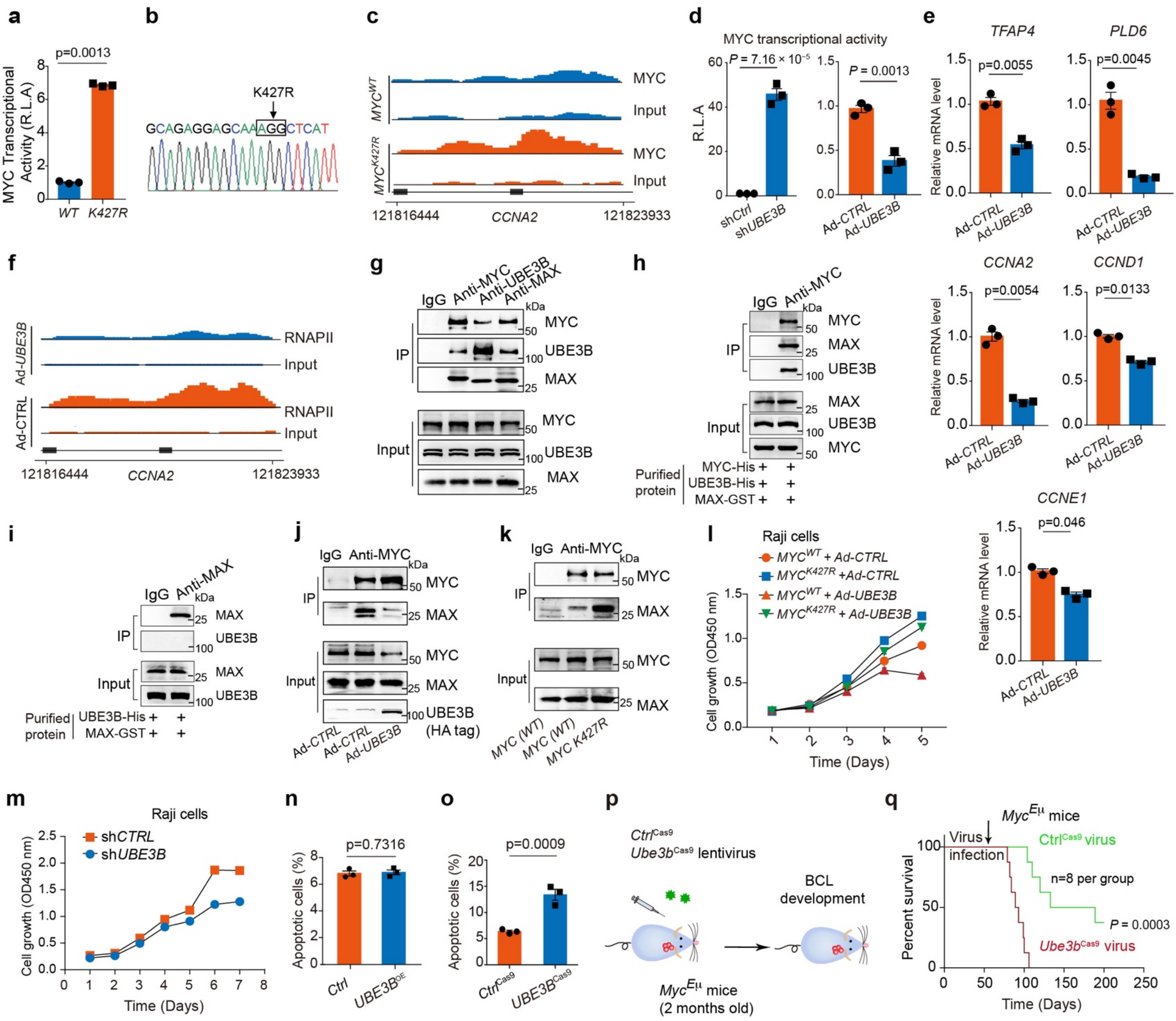

Supplementary Figure 6. UBE3B Inhibits MYC Function to Attenuate Lymphomagenesis. Related to Fig. 4. **(a)** The effects of *K427R* mutation on the transcriptional activity of MYC. HEK 293T cells were transfected with reporter genes and wildtype of *MYC* or *K427R* mutant. After 24 hr of transfection, luciferase activities were measured. Shown is means ± S.E.M of 3 independent experiments. Statistical significance was determined by two-tailed Student's t test. *P* value: 0.0013. (**b**) Sequencing verification of the codon replacement by CRISPR-Cas9 resulting in *MYC (K427R)*. **(c)** ChIP-sequencing tracks for *CCAN2* from indicated groups normalized to spike-in controls. (**d**) The effects of *UBE3B* deletion or overexpression on the transcriptional activity of MYC. sh*Ctrl*, sh*UBE3B*, Ad-*Ctrl*, and Ad-*UBE3B* Raji cells were co-transfected with the reporter genes of MYC transcriptional

activity. After 24 hr of transfection, luciferase activities were measured. Shown is means ± S.E.M of 3 independent experiments. Statistical significance was determined by two-tailed Student's t test. *P* value: 7.16×10^{-5}, 0.0013. **(e)** The mRNA levels of MYC target genes in the indicated cells with or without *UBE3B* overexpression. Shown is means ± S.E.M of 3 independent experiments. Statistical significance was determined by two-tailed Student's t test. *P* value: 0.0055, 0.0045, 0.0054, 0.0133, 0.046. **(f)** ChIP-sequencing tracks for *CCNA2* from indicated cells with or without *UBE3B* overexpression normalized to spike-in controls. **(g)** The heterotrimer of MYC, UBE3B and MAX in Raji cells was detected by CO-IP assays. **(h)** The direct interaction of purified MYC with UBE3B or MAX was detected by CO-IP assays. **(i)** UBE3B did not directly interact with MAX, which was detected by CO-IP assay. **(j)** UBE3B overexpression decreased the interaction of MYC and MAX in Raji cells. **(k)** K427R mutation increased the interaction of MYC and MAX. HEK 293T cells were transfected with MAX and MYC or K427R mutant. After 24 hr of transfection, the interactions were measured by CO-IP assay without Ad-UBE3B infection. (**l**) Cell growth curves of in *MYC*^{WT}*+Ad-CTRL, MYC*^{K427R}*+Ad-CTRL*, *MYC*^{WT}*+Ad-UBE3B,* and *MYC*^{K427R}*+Ad-UBE3B* Raji cells for the indicated times. Shown is means ± S.E.M of 3 independent experiments. (**m**) Cell growth curves of Raji cells with or without *UBE3B* depletion for the indicated times. (**n**) The apoptotic ratio of Raji cells with *UBE3B* deletion via flow cytometry analysis with Annexin V-APC/7-AAD staining. Shown is means ± S.E.M of 3 independent experiments. Statistical significance was determined by two-tailed Student's t test. **(o)** Schematic strategy for studying BCL development in *Myc*^{Eμ} mice with or without infection of lentivirus *Ube3b*^{Cas9}. (**p**) Kaplan-Meier survival curves for *Myc*^{Eμ} mice with or without infection of lentivirus *Ube3b*^{Cas9} (n = 8 per group). Statistical difference was determined by two-sided log-rank test. *P* value: 0.0003.

Source data are provided as a Source Data file.

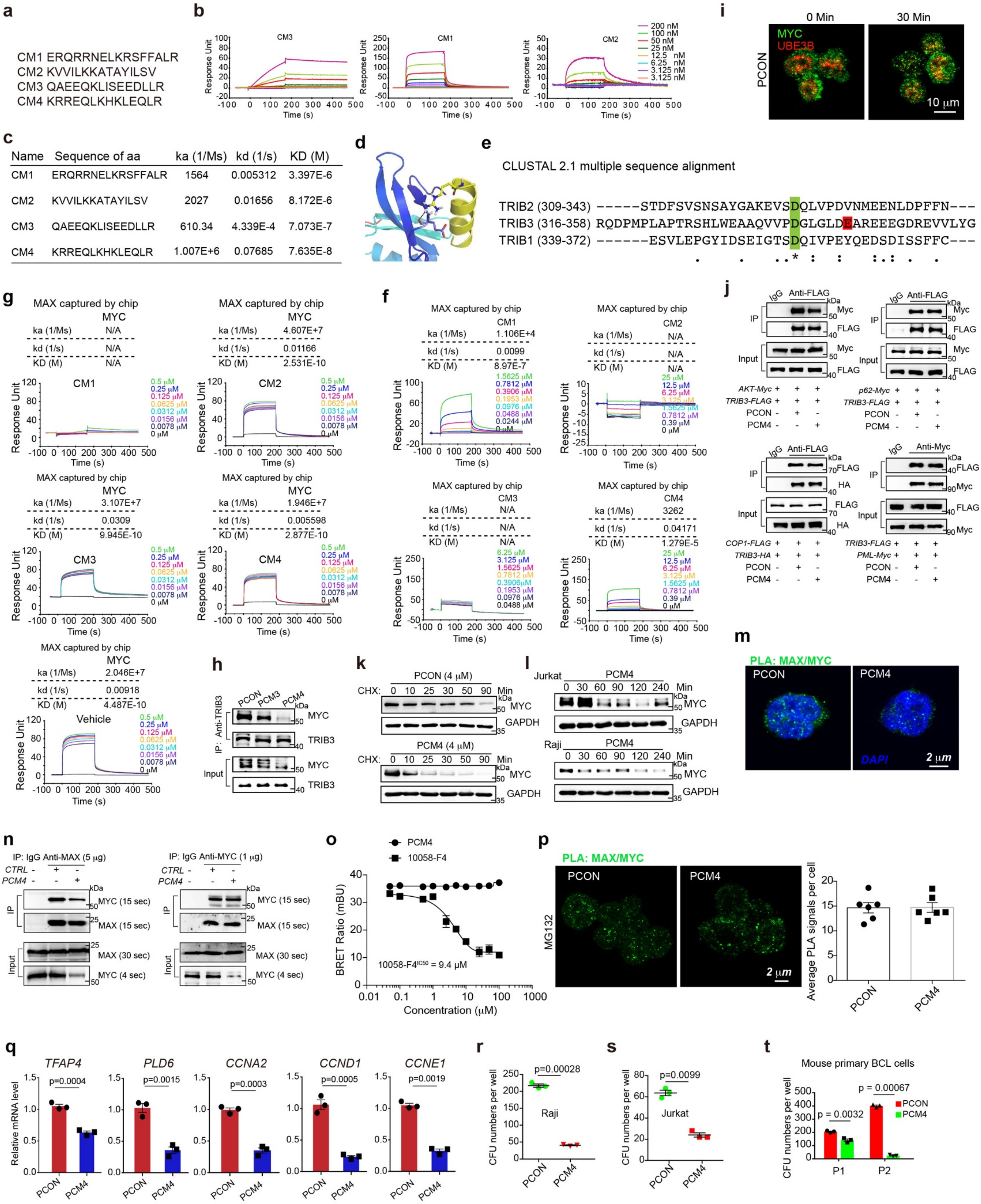

a
CM1 ERQRRNELKRSFFALR
CM2 KVVILKKATAYILSV
CM3 QAEEQKLISEEDLLR
CM4 KRREQLKHKLEQLR
b
CM3
CM1
CM2
Response Unit
Time (s)
200 nM
100 nM
50 nM
25 nM
12.5 nM
6.25 nM
3.125 nM
3.125 nM
c
Name | Sequence of aa | ka (1/Ms) | kd (1/s) | KD (M)
CM1 | ERQRRNELKRSFFALR | 1564 | 0.005312 | 3.397E-6
CM2 | KVVILKKATAYILSV | 2027 | 0.01656 | 8.172E-6
CM3 | QAEEQKLISEEDLLR | 610.34 | 4.339E-4 | 7.073E-7
CM4 | KRREQLKHKLEQLR | 1.007E+6 | 0.07685 | 7.635E-8
d
e
CLUSTAL 2.1 multiple sequence alignment
TRIB2 (309-343) -----STDFSVSNSAYGAKEVSDQLVPDVNMEENLDPFFN---
TRIB3 (316-358) RQDPMPLAPTRSHLWEAAQVVPDGLGLDEAREEEGDREVVLYG
TRIB1 (339-372) ------ESVLEPGYIDSEIGTSDQIVPEYQEDSDISSFFC---
g
MAX captured by chip
MYC
ka (1/Ms) N/A
kd (1/s) N/A
KD (M) N/A
CM1
MAX captured by chip
MYC
ka (1/Ms) 4.607E+7
kd (1/s) 0.01166
KD (M) 2.531E-10
CM2
MAX captured by chip
MYC
ka (1/Ms) 3.107E+7
kd (1/s) 0.0309
KD (M) 9.945E-10
CM3
MAX captured by chip
MYC
ka (1/Ms) 1.946E+7
kd (1/s) 0.005598
KD (M) 2.877E-10
CM4
MAX captured by chip
MYC
ka (1/Ms) 2.046E+7
kd (1/s) 0.00918
KD (M) 4.487E-10
Vehicle
0.5 μM
0.25 μM
0.125 μM
0.0625 μM
0.0312 μM
0.0156 μM
0.0078 μM
0 μM
Response Unit
Time (s)
f
MAX captured by chip
CM1
ka (1/Ms) 1.106E+4
kd (1/s) 0.0099
KD (M) 8.97E-7
MAX captured by chip
CM2
ka (1/Ms) N/A
kd (1/s) N/A
KD (M) N/A
MAX captured by chip
CM3
ka (1/Ms) N/A
kd (1/s) N/A
KD (M) N/A
MAX captured by chip
CM4
ka (1/Ms) 3262
kd (1/s) 0.04171
KD (M) 1.279E-5
h
IP: Anti-TRIB3
Input
PCON PCM3 PCM4
MYC
TRIB3
i
0 Min
30 Min
PCON
MYC
UBE3B
10 μm
j
IgG Anti-FLAG
IP
Input
Myc
FLAG
AKT-Myc
TRIB3-FLAG
PCON
PCM4
p62-Myc
COP1-FLAG
TRIB3-HA
HA
IgG Anti-Myc
TRIB3-FLAG
PML-Myc
k
PCON (4 μM)
PCM4 (4 μM)
CHX: 0 10 25 30 50 90 Min
MYC
GAPDH
l
Jurkat
Raji
PCM4
0 30 60 90 120 240 Min
MYC
GAPDH
m
PLA: MAX/MYC
PCON
PCM4
DAPI
2 μm
n
IP: IgG Anti-MAX (5 μg)
IP: IgG Anti-MYC (1 μg)
CTRL
PCM4
MYC (15 sec)
MAX (15 sec)
MAX (30 sec)
MYC (4 sec)
IP
Input
o
PCM4
10058-F4
BRET Ratio (mBU)
10058-F4IC50 = 9.4 μM
Concentration (μM)
p
PLA: MAX/MYC
PCON
PCM4
MG132
2 μm
Average PLA signals per cell
q
TFAP4
PLD6
CCNA2
CCND1
CCNE1
Relative mRNA level
p=0.0004
p=0.0015
p=0.0003
p=0.0005
p=0.0019
PCON
PCM4
r
p=0.00028
Raji
CFU numbers per well
s
p=0.0099
Jurkat
t
Mouse primary BCL cells
p = 0.00067
p = 0.0032
PCON
PCM4
P1
P2

Supplementary Figure 7. Disturbing the TRIB3/MYC Interaction Destabilizes MYC and Inhibits Lymphoma. Related to Fig. 6. (**a**) The amino acid (aa) sequences of α-helical peptides covering the TRIB3-binding region from MYC are shown. (**b,c**) Kinetic interactions of α-helical peptides and TRIB3 protein were determined by SPR analyses. (**d**) The highest scoring Dock model of the CM4 and TRIB3 complex is shown. (**e**) Sequence alignment within the c terminus of TRIB proteins. (**f**) Kinetic interactions of α-helical peptides and MAX protein were determined by SPR analyses. (**g**) CM1 peptide decreased the binding of MAX and MYC. Kinetic interactions of MAX and MYC protein were determined by SPR analyses with indicated peptides (CM1, CM2, CM3 or CM4). (**h**) Co-IP assays evaluated the effect of PCM4 on the interactions of TRIB3/MYC. Raji cells were treated with 4 μM of the indicated peptide. Six hours later, cell extracts were IP with anti-TRIB3 Ab and blotted with the indicated Abs. (**i**) Co-localization of MYC and UBE3B in Raji cells treated with 4 μM of PCON for 30 min. The data are presented as presentative from 3 independent experiments. (**j**) Co-IP assays evaluated the effect of PCM4 on the interactions of TRIB3/AKT, TRIB3/p62, TRIB3/COP1, TRIB3/PML. The data are presented as presentative from 3 independent experiments. (**k**) MYC degradation in Raji cells treated with CHX (10 μg/mL) and 4 μM of PCON or PCM4 for the indicated times. (**l**) MYC expression in Jurkat and Raji cells treated with 4 μM of PCM4 for the indicated times. (**m**) Co-localization of MYC and MAX was detected in PCM4-treated Raji cells by Duolink PLA assay. Scale bar, 2 μm. (**n**) Effect of PCM4 treatment on MYC/MAX interaction. $4*10^7$ Raji cells were treated with CTRL (PCON) or PCM4 for 4 hr (4 μM). Cell extracts were IP with rabbit immunoglobulin G (IgG), anti-MAX (5 μg, saturating), or anti-MYC (1 μg, non-saturating) Ab and blotted with indicated Ab. (**o**) Dose-response curve against the specific inhibitor 10058-F4 and PCM4 in the MYC-MAX NanoBRET™ screen. NanoBRET™ ratio were tested in a 10-point dose-response experiment in HEK293 cells transiently transfected with MYC- and MAX-fusion constructs. The NanoBRET™ ratio was calculated from raw values for NanoLuc® and HaloTag® signals. IC_{50} value are indicated for 10058-F4. (**p**) Co-localization of MYC and MAX in Raji cells with indicated treatment. Raji cells were treated with PCM4 (4 μM) or PCON for 12hr, and then these cells were pretreated with MG132 (20 μM) for 6 hr and detected by Duolink PLA assay. Scale bar, 2 μm. (**q**) The mRNA levels of *MYC* target genes in the indicated cells with or without PCM4 treatment. Shown is means ± S.E.M of 3 independent experiments. Statistical significance was determined by two-tailed Student's t test. *P* value: 0.0004, 0.0015, 0.0003, 0.0005, 0.0019. (**r,s**) The colony formation numbers of Raji and Jurkat cells with the indicated treatment for 7 days. Shown is means ± S.E.M of 3 independent experiments. Statistical significance was determined by two-tailed Student's t test. *P* value: 0.00028 **r**,

0.0099 **s**. (**t**) PCM4 decreased the serial colony formation of primary mouse BCL cells from *Myc*$^{E\mu}$ mice. Shown is means ± S.E.M of 3 independent experiments. Statistical significance was determined by two-tailed Student's t test. *P* value: 0.0032, 0.00067.

Source data are provided as a Source Data file.

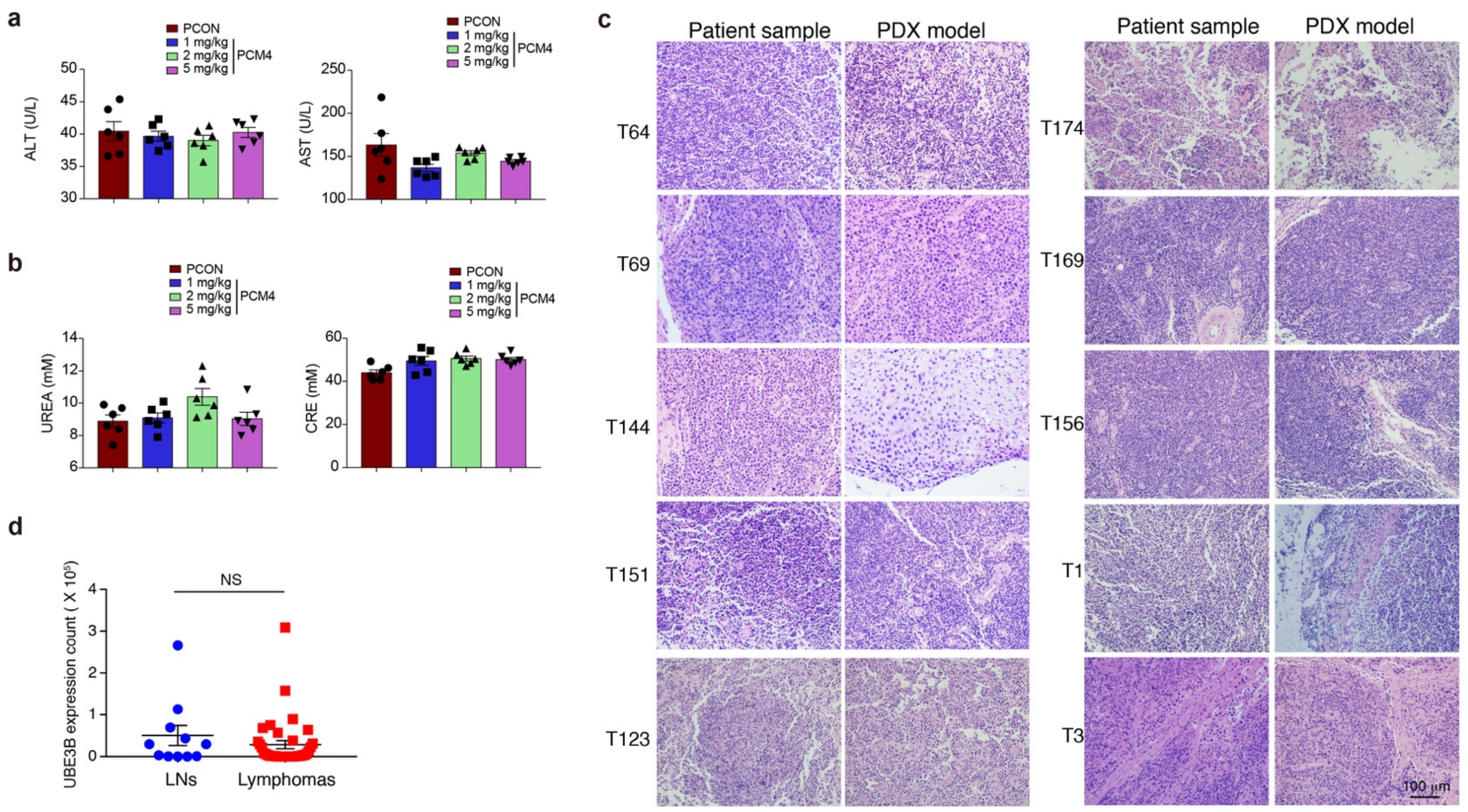

Supplementary Figure 8. No Obvious Toxicity Against Liver/Kidney Function was Observed During PCM4 Treatment; Clinical Characteristics of the PDX Model and Lymphoma Patients. Related to Fig. 7 and 8. (**a**) The serum alanine transaminase (ALT) and aspartate transaminase (AST) levels of *Myc*$^{E\mu}$ mice with the indicated treatment were detected to evaluate liver function (n = 6 per group). Data are represented as means ± SEM. (**b**) The serum UREA and creatinine (CRE) levels of *Myc*$^{E\mu}$ mice with the indicated treatment were detected to evaluate renal function (n = 6 per group). Data are represented as means ± SEM. (**c**) Hematoxylin and eosin (HE) staining results of tumor tissues from lymphoma patients and the PDX model. Data are represented as representative f 3 independent experiments. Scale bar, 100 μm. (**d**) Statistical analyses of IHC staining of UBE3B in human lymphomas and LN tissues (LNs: n = 11; lymphomas: n = 37). Data are represented as means ± SEM. Statistical significance was determined by two-tailed Student's t test.

Source data are provided as a Source Data file.

Supplementary Table 1. Clinical information of the lymphoma patients (61 men and 29 women) between the ages of 14 and 82.

No.	Diagnosis	Sample origin	Disease status	IHC staining
T64	Diffuse large B-cell lymphoma (DLBCL)	Armpit lymph nodes	Untreated	CD3 (-), CD20 (+), CD5 (+), CD10 (+), Bcl-2 (+), Bcl-6 (+), CD21 (+), CD23 (+), CyclinD1 (-), c-Myc (+)
T69	Diffuse large B-cell lymphoma (DLBCL)-GCB	Cervical lymph nodes	Untreated	CD3 (-), CD20 (strong+), CD5 (+), CD30 (-), Bcl-2 (+), Bcl-6 (+), CD10 (+), c-Myc (+)
T144	Non-Hodgkin lymphoma-TCL (PTCL)	Left supraclavicular lymph nodes	Untreated	CD3 (+), CD4 (+), CD5 (+), CD20 (-), CD79a (-), Mum-1 (+), Bcl-2 (+), CD10 (-), c-Myc (strong+)
T151	Follicular lymphoma (FL)	Inguinal lymph nodes	Untreated	CD3 (+), CD43 (+), PAX5 (+), CD20 (+), CD38 (-), Bcl-2 (+), Bcl-6 (+), CD10 (+), c-Myc (-)
T123	T-cell lymphoblastic lymphoma (T-LBL)	Cervical lymph nodes	Untreated	CD3 (+), CD20 (-), CD5 (+), CD21 (+), PaX-5 (-), CD23 (-), TdT (+), Mum-1 (-), CD10 (+), Bcl-6 (-), CD34 (-), c-Myc (strong+)

T156	Diffuse large B-cell lymphoma (DLBCL-non GCB)	Inguinal lymph nodes	Untreated	CK (-), CD3 (-), CD20 (+), CD10 (-), PaX-5 (+), Mum-1 (+), Bcl-2 (+), Bcl-6 (-), S-100 (-), c-Myc (-)
T174	Hgh grade Follicular lymphoma (FL) - DLBCL	Inguinal lymph nodes	Untreated	CD20 (+), CD79a (+), CyclinD1 (-), CD10 (-), CD5 (weak+), Mum-1 (+), Bcl-6 (-), S-100 (-), c-Myc (+)
T177	Non-Hodgkin lymphoma-BCL	Cervical lymph nodes	Untreated	CD20 (strong+), PaX-5 (strong+), Mum-1 (+), Bcl-2 (+), Bcl-6 (-), CD10 (-), CyclinD1 (-), CD38 (-), AE1/AE3 (-), c-Myc (-)
T169	Diffuse large B-cell lymphoma (DLBCL)	Cervical lymph nodes	Untreated	CD3 (-), CD20 (+), CD5 (-), CyclinD1 (-), Mum-1 (+), Bcl-2 (+), Bcl-6 (+), CD10 (-), c-Myc (+)
T105	Diffuse large B-cell lymphoma (DLBCL-GCB)	Inguinal lymph nodes	Untreated	AE1/AE3 (-), Vimentin (+), S-100 (-), CD30 (+), LCA (+), CD3 (part +), CD20 (+), CD79a (+), ALK (-), Mum-1 (+), Bcl-6 (+), CD10 (-), c-Myc (+)
T104	Diffuse large B-cell lymphoma (DLBCL-GCB)	Cervical lymph nodes	Untreated	LCA (+), AE1/AE3 (-), S-100 (-), CD3 (-), CD20 (+), CD5 (-), CD79a (+), CD4 (-), CD30 (+), CyclinD1 (-), Mum-1 (+), Bcl-6 (+), CD10 (-), c-Myc (+)

T1	Diffuse large B-cell lymphoma (DLBCL-GCB)	Lymph nodes	Untreated	Bcl-6(+), CD10(-), CD117(-), CD20(+), CD3(-), CD45RO(-), c-Myc (-)
T2	Diffuse large B-cell lymphoma (DLBCL-GCB)	Ileum	Untreated	AE1/AE3 (-), Vimentin (+), S-100 (-), CD30 (+), LCA (+), CD3 (part +), CD20 (+), Bcl-6 (+), CD10 (-), c-Myc (-)
T3	T-cell lymphoblastic lymphoma	the left inguinal lymph node	Untreated	CD3 (+), CD20 (-), CD5 (+), CD21 (+), PaX-5 (-), CD23 (-), TdT (+), Mum-1 (-), CD10 (+), Bcl-6 (+), CD34 (-), c-Myc (-)
T4	Burkitt lymphoma	Left neck	Untreated	CD20 (+), CD10 (+), CD79a (+), bcl-6 (+), Ki67 (+, 100%), c-Myc (+)
T5	Burkitt lymphoma	Right neck	Untreated	Bcl-2(+), Bcl-6(-), CD10(-), CD20(+), CD3(-), CD5(+/-), MUM-1(+), c-Myc (+), Ki67 (+, 100%)

WXH-1	Mantle cell lymphoma (MCL)	The right armpit	Untreated	C20(+),CD30(-),CD5(+),CD56(-),CD79a(+),Mum(+), c-Myc (+)
L1	B-cell lymphoma (BCL)	the right ureter	NA	Bcl-6(+), CD10(-), CD117(-), CD20(+), LCA(+), Mum-1(+), c-Myc (+)
L2	Diffuse large B-cell lymphoma (DLBCL-GCB)	Armpit	NA	Bcl-2(+), Bcl6(+/-), CD20(+), CD3(-), Ki67(+40%), c-Myc (+)
L3	Diffuse large B-cell lymphoma (DLBCL-GCB)	Left testicle	NA	Bcl-6(+), CD10(-), CD117(-), CD20(+), CD3(-), CD45RO(-), c-Myc (+)
L4	Marginal zone lymphoma (MZL)	Left supraclavicular lymph nodes	NA	Bcl-2(+), Bcl-6(-), CD10(-), CD20(+), Mum(+), Pax-5(+), c-Myc (-)
L5	Mantle cell lymphoma (MCL)	Lymph nodes	NA	C20(+),CD30(-),CD5(+),CD56(-),CD79a(+),Mum(+), c-Myc (-)

L6	Diffuse large B-cell lymphoma (DLBCL)	Ileum	NA	Bcl-2(+), Bcl-6(+/-), CD10(-), CD20(+), CD23(-), CK(-)
L7	Angioimmunoblastic T-cell lymphoma (AITL)	the left inguinal lymph node	NA	ALK(-), Bcl-2(-), Bcl-6(+), CD10(+), CD15(-), CD20(-), CD5(+), c-Myc (-)
L8	T-cell lymphoblastic lymphoma	Left neck	NA	Bcl-2(+), Bcl-6(-), CD10(-), CD3(+), CD15(-), CD34(+), TclT(+/-), c-Myc (-)
L9	Diffuse large B-cell lymphoma (DLBCL)	Right neck	NA	Bcl-2(+), Bcl-6(+), CD10(-), CD20(+), CD21(-), Mum-1(+), c-Myc (+)
L10	Diffuse large B-cell lymphoma (DLBCL)	The right armpit	NA	Bcl-6(+), CD10(-), CD20(+), CD21(-), Pax-5(+), c-Myc(-), c-Myc (+)
L11	Diffuse large B-cell lymphoma (DLBCL)	Neck	NA	Bcl-2(+), Bcl-6(+), CD10(-), CD20(+), CD23(+/-), CD5(-), c-Myc (+)

L12	Diffuse large B-cell lymphoma (DLBCL)	the right ureter	NA	Bcl-2(+), LCA(+), CD56(-), CD99(-), Ki67(+95%), c-Myc (+)
L13	Diffuse large B-cell lymphoma (DLBCL)	Ovary	NA	Bcl-6(+), CD10(+), CD117(-), CD20(+), CK(-), pax-5(+), c-Myc (+)
L14	Diffuse large B-cell lymphoma (DLBCL)	Left neck, armpit	NA	Bcl-2(+), Bcl-6(-), CD10(-), CD15(-), CD79a(+), c-Myc (+)
L15	Angioimmunoblastic T-cell lymphoma (AITL)	Neck	NA	Alk(-), Bcl-2(-), Bcl-6(-), CD30(-), CD5(+), CK(-), c-Myc (-)
L16	Follicular lymphoma (FL)	Right groin	NA	Bcl-2(+), CD20(+), CD3(-), CD5(-), CydinD1(+), Mum-1(-), c-Myc (-)
L17	Follicular lymphoma (FL)	Right neck	NA	Bcl-2(+), Bcl-6(-), CD10(-), CD20(+), Mum-1(+), c-Myc(-)
L18	T-cell lymphoblastic lymphoma	Right neck	NA	Bcl-2(-), Bcl-6(-), CD10(-), CD5(+), TclT(+), CD45RO(+/-), c-Myc (+)
L19	Diffuse large B-cell lymphoma (DLBCL)	Neck	NA	Bcl-2(-/+), Bcl-6(+/-), CD10(-), CD20(+), CD21(-), CD3(-）, c-Myc (+)

L20	Diffuse large B-cell lymphoma (DLBCL)	Left neck	NA	Bcl-2(-), Bcl-6(+), CD10(+), CD20(+), CD3(-), CD5(-), c-Myc (+)
L21	Diffuse large B-cell lymphoma (DLBCL)	Left neck	NA	CD20(+), MUM-1(+), Bcl-l(+/-), CD10(-), CD3(-), c-Myc (+)
L22	Diffuse large B-cell lymphoma (DLBCL)	Spleen	NA	Bcl-2(-), Bcl-6(-), CD10(+), CD20(+), CD3(-), CD38(-), c-Myc (+)
L23	Diffuse large B-cell lymphoma (DLBCL)	gum	NA	Bcl-2(+), Bcl-6(-), CD10(-), CD20(+), CD3(-), CD792(+/-), CK(-), c-Myc (-)
L24	Mantle cell lymphoma (MCL)	Cervical lymph nodes	NA	Bcl-2(+), Bcl-6(-), CD10(-), CD20(+), CD3(-), CD5(+/-), MUM-1(+), c-Myc (+)
L25	non-Hodgkinlymphoma (B cell)	left testicle	NA	Bcl-2(+), Bcl-6(+), CD10(-), CD117(-), CD20(+), CD79a(+), c-Myc (+)
L26	Diffuse large B-cell lymphoma (DLBCL)	Ileocecus	NA	Bcl-6(-), CD10(-), CD20(+), CD23(-), ki67(70%+), c-Myc (+)
L27	NK/T cell lymphoma NKTCL	Left testicle	NA	Bcl-2(+), Bcl-6(-), CD10(-), CD117(-), CD43(+), c-Myc (+)
L28	Angioimmunoblastic T-cell lymphoma (AITL)	Groin	NA	CD20(B cell+), CD23(-), CD3(+), CD30(+), EMA(-), c-Myc (+)

L29	Marginal zone B-cell lymphoma (MZBL)	deep neck	NA	Mum(+), CD20(+), CK(-), Bcl-6(+), CD23(+), CD5(-), c-Myc (-)
L30	Angioimmunoblastic T-cell lymphoma (AITL)	Cervical lymph nodes	NA	Bcl-2(+), CD10(+/-), CD21(+), CD3(+), CyclinD1(-), c-Myc (-)
L31	Angioimmunoblastic T-cell lymphoma (AITL)	Cervical lymph nodes	NA	Bcl-6(+), CD10(+), CD15(-), CD34(+), CD79a(-), Ki67(80%+), c-Myc (+)
L32	Diffuse large B-cell lymphoma (DLBCL)	Right neck	NA	Bcl-2(-), Bcl-6(+), CD10(+), CD20(+), CD23(-), CK(-), c-Myc (+)
L33	Diffuse large B-cell lymphoma (DLBCL)	Right colon	NA	Bcl-2(+), Bcl-6(-), CD10(-), CD20(+), CD23(-), C56(-), c-Myc (-)
L34	Burkitt lymphoma	Submandibular	NA	Bcl-2(-), Bcl-6(+), CD10(+), CD15(-), CD20(+), CD43(+), c-Myc (-)
L35	Diffuse large B-cell lymphoma (DLBCL)	Left adnexa	NA	CD117(-), LCA(+), Bcl-6(+), CD3(-), EMA(-), Vimintin(+), c-Myc (+)

L36	Diffuse large B-cell lymphoma (DLBCL)	Right jaw	NA	Bcl-2(+), Bcl-6(+), CD10(-), CD20(+), CD23(-), Mum-1(+), c-Myc (+)
L37	Diffuse large B-cell lymphoma (DLBCL)	Small intestine	NA	CD20(+), CD3(-), LCA(+), CD56(-), P40(-), CK20(-), c-Myc (-)
L38	Diffuse large B-cell lymphoma (DLBCL)	Spleen	NA	CD10(-), CD20(+), CD23(-), CD79a(+/-), MUM-1(+), CyclinD1(-), c-Myc (-)
L39	Mantle cell lymphoma (MCL)	Left neck	NA	Bcl-2(+), Bcl-6(-), CD10(-), CD20(+), CD79a(+), CydinD1(+), c-Myc (-)
L40	Diffuse large B-cell lymphoma (DLBCL)	Right testis	NA	Bcl-2(+), CD10(+), CD117(-), CD20(+), CD3(-), CK(-), MUM-1(+), c-Myc (+)
L4166	Diffuse large B-cell lymphoma (DLBCL)	Jaw	NA	Bcl-6(+), CD20(+), CD21(-), CD3(-), CD30(-),CD10(+/-), Ki67(+70%), MUM(+), c-Myc (-)
L4270	Mantle cell lymphoma (MCL)	Neck	NA	Bcl-2(+), Bcl-6(-), CD10(-), CD20(+), CD21(+), CD3(-), CyclinD1(+), MUM(-), c-Myc (+)

L4488	Mantle cell lymphoma (MCL)	The left inguinal lymph node	NA	CD15(-), Bcl-2(+), Bcl-6(+/-), CD10(-), CD20(+), CD21(-), CD3(-), CD5(+), CD43(+), c-Myc (+)
L4717	Diffuse large B-cell lymphoma (DLBCL)	Right testis	NA	Bcl-6(+), CD10(-), CD20(+), CD3(-), CD79a(+), CK(-), MUM-1(+), PLAP(-), c-Myc (+)
L7020	Diffuse large B-cell lymphoma (DLBCL)	The left hemorrhage of basal ganglion	NA	Bcl-6(+), CD10(-), CD20(+), CD3(-), CD56(-), CyclinD1(-), GFAP(-), Ki67(+40％), c-Myc (-)
L7023	Diffuse large B-cell lymphoma (DLBCL)	Stomach	NA	CD10(focal area+), CD20(+), CD21(-), CD3(-), CK(-), CyclinD1(-), MUM-1(+), Syn(-), c-Myc (+)
L7404	non-Hodgkinlymphoma (B cell)	The left inguinal lymph node	NA	Bcl-2(+), CD10(-), CD20(+), CD21(FDC+), CD3(-), CD30(+), CD79a(+), CyclinD1(-), c-Myc (-)
L15656	B-cell lymphoma (BCL)	Right belly	NA	Bcl-6(+/-), CD10(-), CD20(+), CD21(-), CD23(-), CD3(+/-), CD5(+/-), MUM-1(-), c-Myc (-)

L1514	Diffuse large B-cell lymphoma (DLBCL)	Right breast	NA	CD20(+), CD3(-), LCA(+), CD56(-), CK7(-), CgA(-), CK(-), CD38(-), CK10(-), Ki67(+), c-Myc (-)
L2066	Diffuse large B-cell lymphoma (DLBCL)	Neck	NA	Bcl-6(+), CD10(-), CD20(+), CD21(-), CD3(-), Ki67(+80%), Pax-5(+), c-Myc (-)
L2108	T-cell lymphoma (TCL)	Jaw	NA	Bcl-2(-), CD20(-), CD21(-), CD3(+), CD43(+), CD56(+), CK(+), CyclinD1(+), c-Myc (-)
L2129	Diffuse large B-cell lymphoma (DLBCL)	Abdominal wall	NA	Bcl-6(+), CD10(+), CD20(+), CD21(-), CD23(-), CD3(-), Ki67(+60%), MUM-1(+), c-Myc (-)
L2662	T-cell lymphoma (TCL)	Inguinal lymph nodes	NA	Bcl-6(+), CD10(-), CD20(+), CD3(+), CD21(+), CyclinD1(+/-), MUM-1(+/-), c-Myc (+)
L2834	B-cell lymphoma (BCL)	Neck	NA	Bcl-2(+), Bcl-6(-), CD10(-), CD20(+), CD21(+), CD23(+), CD3(-), MUM-1(+), c-Myc (+)
L8529	Diffuse large B-cell lymphoma (DLBCL)	Abdomen	NA	Bcl-2(+), Bcl-6(-), CD10(-), CD23(-), CD3(-), CD30(-), CD5(-), CyclinD1(-), Ki67(+), c-Myc (-)

L9049	B-cell lymphoma (BCL)	Right testis	NA	CD20(-), CD3(-), CD43(+), MPO(-), TdT(+), CD45RO(-), Pax-5(+), Ki67(+), c-Myc (+)
L9495	B-cell lymphoma (BCL)	Stomach	NA	Bcl-2(-), Bcl-6(-), CD10(-), CD20(+), CD21(+), CD3(-), CD35(-), Ki67(+ < 30%), c-Myc (-)
L12381	Mantle cell lymphoma (MCL)	Colon	NA	Bcl-6(-), CD10(-), CD20(+), CD23(-),CD3(-), CD5(-), CD79a(+/-)
L12962	Diffuse large B-cell lymphoma (DLBCL)	The left inguinal lymph node	NA	Bcl-6(+), CD10(-), CD20(+), EMA(+), Ki67(+80%), CD30(+), NSE(-), c-Myc (+)
L12477	Plasmablastic lymphoma (PBL)	Left kidney	NA	Bcl-2(-), Bcl-6(-), CD10(-), CD138(-), CD20(-), CD38(+)CD34(-), CD3(-), CD30(-), Lanbda(+), MUM-1(+), CD4(-), MPO(-), c-Myc (+)
L9011	Diffuse large B-cell lymphoma (DLBCL)	Spleen	NA	Bcl-6(+), CD10(-), CD20(+), EMA(+), Ki67(+80%), CD30(+), NSE(-), c-Myc (+)

L11450	Mantle cell lymphoma (MCL)	Groin	NA	CD20(+), Bcl-2(+), CD79a(+), CyclinD1(+), Ki67(+ <10%), CD3(-), CD5(-), CD21(-), c-Myc (+)
L11502	Diffuse large B-cell lymphoma (DLBCL)	Left eyelid	NA	CD20(+), CD79a(+), CD43(+), CD10(+), Bcl-2(+), Bcl-6(+), Ki67(+ >60%), c-Myc (+)
L11658	Diffuse large B-cell lymphoma (DLBCL)	Left jaw	NA	CD20(+), Bcl-6(+), MUM(+), CK(-), Syn(-), CD56(-), CD3(-), CD5(-), c-Myc (-)
L13669	Diffuse large B-cell lymphoma (DLBCL)	Stomach	NA	CK(-),CD20(+), CD79a(+), CD3(-), CD35(-), CD21(-), CD10(+), Bcl-6(+), c-Myc (+)
L14456	Diffuse large B-cell lymphoma (DLBCL)	Thoracic spinal canal	NA	Bcl-6(+), CD10(+), CD138(-),CD20(+), CD21(-), CD3(-), Ki67(+40%), MUM(+), c-Myc (-)
L15300	Diffuse large B-cell lymphoma (DLBCL)	Belly	NA	Bcl-2(+), Bcl-6(+/-), CD10(+), CD20(+), CD21(-), CD23(-), CD3(-), CD5(-), c-Myc (-)
L13428	Diffuse large B-cell lymphoma (DLBCL)	Perineum	NA	CD20(+), CD3(-), CD30(-), CD43(-), CD56(-), CK(-), Ki67(+90%), TLA(-), c-Myc (+)

L14788	Diffuse large B-cell lymphoma (DLBCL)	Right parotid gland	NA	Bcl-6(+), CD10(+), CD138(-),CD20(+), CD21(-), CD3(-), Ki67(+40%), MUM(+), c-Myc (+)
L15678	Mantle cell lymphoma (MCL)	Ileocecum	NA	Bcl-6(+), CD10(-), CD20(+), CD23(-), CD3(-), CD5(+/-), CD79a(+), CyclinD1(+), c-Myc (-)
L16406	Diffuse large B-cell lymphoma (DLBCL)	Neck	NA	CD35(+), MPO(-), CD43(+), CD3(-), CD20(+), CD21(+),CD5(-), CD10(-), Bcl-2(+), Bcl-6(-), c-Myc (+)
L16547	Diffuse large B-cell lymphoma (DLBCL)	Left inguinal	NA	Bcl-6(+), CD10(-), CD20(+), CD3(-), CD79a(+/-), CyclinD1(-), MUM-1(+), Ki67(+), c-Myc (-)
L2231	Diffuse large B-cell lymphoma (DLBCL)	Spleen	NA	Bcl-6(+), CD30(+), CD5(-), CD56(-), CK(-), CyclinD1(-), MUM-1(+), C-Myc (+)

Abbreviation: NA, not applicable; R, rearrangement; IHC, immunohistochemistry; BM, bone marrow; NEG, negative; POS, positive; GCB, germinal center B-cell–like

Supplementary Table 2. Sequencing the coding region of *TRIB3* and *UBE3B* in human lymphoma samples

Sample no	*UBE3B*		*TRIB3*			c-Myc status (IHC)
	cds (1037) exon12 (97)	cds (2886) exon26 (76)	exon1 Protein (84)	exon2 Protein (111)	exon3 Protein (323)	
T174	G/G	C/C	A/G	C/C	C/T	+
T144	G/G	C/C	A/A	C/C	C/T	+
T177	G/G	C/C	A/A	C/C	C/T	–
T69	G/A	C/T	A/A	C/C	C/T	+
T64	G/A	C/C	A/A	T/T	C/T	+
T169	G/A	C/C	A/A	C/C	C/T	+
T151	G/G	C/C	A/G	C/C	C/T	–
T123	G/A	C/C	A/A	C/C	C/T	+
T156	G/A	C/T	A/A	C/T	C/T	–
L1	G/A	C/C	A/A	C/C	C/T	+
L2	A/A	C/C	A/G	C/C	T/T	+
L3	G/A	C/C	A/G	T/T	C/C	+
L4	G/G	C/T	A/G	C/C	C/C	–
L5	G/G	C/C	A/A	C/C	C/C	–
L6	G/G	C/C	A/A	T/T	C/C	+
L7	G/G	C/C	A/A	C/C	C/T	–
L8	G/G	C/C	A/G	C/C	C/T	–
L9	A/A	C/C	A/A	C/C	C/T	+
L10	A/A	C/T	A/G	C/C	C/C	+
L11	G/G	C/C	A/G	C/C	C/T	+
L12	G/A	C/C	A/G	C/C	C/T	+
L13	A/A	C/T	A/A	C/T	C/T	+
L14	G/A	C/C	A/A	C/C	C/T	–
L15	G/G	C/C	A/A	C/C	T/T	–
L16	A/A	C/C	A/A	C/C	C/C	–
L17	G/G	C/C	A/G	C/C	C/C	–
L18	G/A	C/C	A/A	C/C	C/C	+
L19	G/A	C/C	A/G	C/C	C/C	+
L20	G/A	C/C	A/A	C/C	C/C	+
L21	G/A	C/T	A/A	C/C	C/T	+
L22	G/A	C/C	A/A	C/C	T/T	+

L23	G/G	C/C	A/G	C/C	C/T	–
L24	G/A	C/C	A/A	C/C	C/C	+
L25	A/A	C/C	A/G	C/C	C/C	+
L26	G/A	C/C	A/G	C/C	C/C	+
L27	G/G	C/C	A/G	C/C	C/C	+
L28	G/G	C/C	A/G	C/C	C/C	+
L29	G/G	C/C	A/G	C/C	C/T	–
L30	G/A	C/C	A/A	C/C	T/T	–
L31	A/A	C/C	A/A	C/C	T/T	+
L32	G/G	C/C	A/A	C/T	C/T	+
L33	G/G	C/C	A/A	T/T	C/C	–
L34	G/G	C/C	A/A	C/T	C/C	–
L35	G/A	C/C	A/A	C/C	C/T	+
L36	G/G	C/C	A/G	C/T	C/C	+
L37	G/A	C/C	A/A	T/T	C/C	–
L38	G/G	C/C	A/A	C/T	C/C	–
L39	G/G	C/C	A/A	C/T	C/C	–
L40	G/G	C/C	A/G	C/C	C/T	+
T1	G/A	C/C	A/A	C/C	C/C	–
T2	G/G	C/C	A/A	C/C	C/T	–
T3	G/A	C/C	A/A	C/T	C/C	–
T4	G/G	C/C	A/G	C/C	C/T	+
T5	G/G	C/C	A/G	C/T	C/C	+
WXH-1	G/G	C/C	A/A	C/T	C/C	–
WXH-2	G/A	C/T	A/A	C/C	C/T	+
L4166	G/G	C/C	A/A	C/C	C/C	–
L4270	G/A	C/C	A/A	C/C	C/C	+
L4488	G/A	C/C	A/A	C/C	C/C	+
L4717	G/A	C/C	A/G	C/C	C/C	+
L7020	G/G	C/C	A/A	C/C	C/T	–
L7023	G/A	C/C	A/A	C/T	T/T	+
L7404	G/G	C/C	A/A	C/T	C/C	–
L15656	G/G	C/C	A/A	C/T	C/C	–
L1514	G/G	C/C	A/A	C/C	C/C	–
L2066	G/A	C/C	A/A	C/C	T/T	–
L2108	G/G	C/T	A/A	C/C	C/C	–

L2129	G/G	C/C	A/A	C/C	C/C	–
L2662	G/A	C/C	A/A	C/C	C/C	+
L2834	G/A	C/C	A/A	C/C	C/C	+
L8529	G/A	C/C	A/A	C/C	C/C	–
L9049	G/G	C/C	A/G	C/C	C/C	+
L9495	G/G	C/C	A/A	C/C	C/T	–
L12381	G/G	C/C	A/G	C/C	C/C	+
L12962	G/G	C/C	A/G	C/C	C/T	+
L12477	G/G	C/C	A/G	C/C	C/C	–
L9011	G/G	C/C	A/G	T/T	C/T	+
L11450	G/A	C/C	A/G	C/C	C/C	+
L11502	G/A	C/C	A/G	C/C	C/C	+
L11658	G/G	C/C	A/G	C/C	C/C	–
L13669	G/A	C/T	A/A	C/T	C/C	+
L14456	G/G	C/C	A/A	C/T	C/C	–
L15300	G/G	C/C	A/G	C/C	C/C	–
L13428	G/G	C/T	A/G	C/C	C/C	+
L14788	G/A	C/C	A/A	C/C	C/C	+
L15678	G/G	C/C	A/A	C/T	C/C	–
L16406	G/A	C/T	A/G	C/C	C/C	+
L16547	G/A	C/C	A/A	C/T	C/C	–
L2231	G/A	C/C	A/G	C/T	C/T	+
	Missense R346Q	Synonymous	Missense Q84R	Synonymous	Synonymous	
	cGg/cAg	tcC/tcT	cAg/cGg	taC/taT	gcC/gcT	
	R346Q	S962S	Q84R	Y111Y	A323A	

Supplementary Table 3. Co-IP assay to identify potential interacting proteins of c-Myc

Types of E3s	Ligase (source)	Also Known As	Result of Co-IP assay
RING	C-CBL (Sino Biological Inc.)	CBL2; NSLL; C-CBL; RNF55; FRA11B	-
	AMFR (Sino Biological Inc.)	GP78; RNF45	-
	CBLL1 (Sino Biological Inc.)	HAKAI; RNF188	-
	CHFR (Sino Biological Inc.)	RNF116; RNF196	-
	LNX1 (Sino Biological Inc.)	LNX; MPDZ; PDZRN2	-
	MGRN1 (Sino Biological Inc.)	RNF156	-
	RNF4 (Sino Biological Inc.)	SLX5; SNURF; RES4-26	+
	MARCH1 (Sino Biological Inc.)	RNF171; MARCH1	-
	BIRC7 (Sino Biological Inc.)	KIAP; LIVIN; MLIAP; RNF50; ML-IAP	-
	IDOL (ORIGENE Inc.)	MIR，MYLIP	-
	TRIM10 (ORIGENE Inc.)		-
	BRCA1 (Sino Biological Inc.)		-
	MDM2 (Sino Biological Inc.)		-
	MIB1 (Sino Biological Inc.)	MIB; DIP1; ZZZ6; DIP-1; LVNC7; ZZANK2	-

	MIB2 (Sino Biological Inc.)	ZZZ5; ZZANK1	-
	RCHY1 (Sino Biological Inc.)	ZCHY; ARNIP; CHIMP; PIRH2; RNF199; ZNF363; PRO1996	-
	MYCBP2	PAM; Phr; Myc-bp2	+
	PJA1 (Sino Biological Inc.)	RNF70; PRAJA1	-
	RFLL (Sino Biological Inc.)	CARP2; FRING; CARP-2; RNF189; RNF34L; RIFIFYLIN	-
	COP1 (Sino Biological Inc.)	RFWD2; RNF200	+++
	RNF5 (Sino Biological Inc.)	RMA1; RING5	-
	RNF8 (Sino Biological Inc.)		-
	RNF15 (Sino Biological Inc.)	TRIM38; RORET	-
	RNF20 (Sino Biological Inc.)	BRE1; BRE1A;hBRE1	-
	RNF34 (Sino Biological Inc.)	RFI; RIF; RIFF; hRFI; CARP1; CARP-1	-
	RNF40 (Sino Biological Inc.)	BRE1B; RBP95; STARING	-
	RNF81 (ORIGENE Inc.)	TRIM21 ; SSA; RO52; SSA1; Ro/SSA	++
	RNF89 (Sino Biological Inc.)	TRIM6	++
	RNF125 (Sino Biological Inc.)	TNORS; TRAC1; TRAC-1	-

	RNF128 (Sino Biological Inc.)	GRAIL	-
	RNF138 (Sino Biological Inc.)	NARF; HSD-4; STRIN; hNARF	-
	RNF168 (Sino Biological Inc.)		-
	TRIM32 (Sino Biological Inc.)	HT2A; BBS11; TATIP; LGMD2H; LGMDR8	+++
	SHPRH (Sino Biological Inc.)		-
	SIAH1 (Sino Biological Inc.)	SIAH1A	-
	SIAH2 (Sino Biological Inc.)		-
	PELI1 (Sino Biological Inc.)		-
	PELI2 (Sino Biological Inc.)		-
	TOPORS (Sino Biological Inc.)	LUN; RP31; P53BP3; TP53BPL	-
	TRAF6 (Sino Biological Inc.)	RNF85; MGC:3310	+
	TRAF7 (Sino Biological Inc.)	RFWD1; RNF119	-
	TRIM63 (Sino Biological Inc.)	IRF; SMRZ; MURF1; MURF2; RNF28	-
	UBR1 (Sino Biological Inc.)	PTR1	-
	UBR2 (Sino Biological Inc.)	C6orf133; bA49A4.1; dJ242G1.1; dJ392M17.3	-
	UHRF2 (Sino Biological Inc.)	NIRF; URF2; RNF107; TDRD23	-

	ZNRF1 (Sino Biological Inc.)	NIN283	-
	UBE4B (Sino Biological Inc.)		-
cullin-RING-based E3 ubiquitin-protein ligase (CRLs)	BTRC (Sino Biological Inc.)	FWD1; FBW1A; FBXW1; bTrCP; FBXW1A; bTrCP1; betaTrCP; BETA-TRCP	-
	FBXW7 (Sino Biological Inc.)	AGO; CDC4; FBW6; FBW7; hAgo; FBX30; FBXW6; SEL10; hCdc4; FBXO30; SEL-10	+++
	SKP2 (Sino Biological Inc.)	p45; FBL1; FLB1; FBXL1	+++
	RBX1 (Sino Biological Inc.)		-
	VHL (Sino Biological Inc.)	RCA1; VHL1; pVHL; HRCA1	+
Other multisubunit E3s	Cdc20 (Sino Biological Inc.)		-
	Cdh1 (Sino Biological Inc.)		-

NEDD4 family (ww domin)	NEDD4 (Sino Biological Inc.)	RPF1;	-
	NEDD4L (Sino Biological Inc.)	RSP5; PVNH7; NEDD4-2; NEDD4.2; hNEDD4-2	-
	SMURF1 (Sino Biological Inc.)		-
	SMURF2 (Sino Biological Inc.)		-

	WWP1 (Sino Biological Inc.)	AIP5; Tiul1; hSDRP1	-
	WWP2 (Sino Biological Inc.)	AIP2; WWp2-like	-
	ITCH (Sino Biological Inc.)	AIF4; AIP4; ADMFD;NAPP1	+
	HECW1 (Sino Biological Inc.)	NEDL1	-
	HECW2 (Sino Biological Inc.)	NEDL2; NDHSAL	-
Other HECTs	UBE3A (ORIGENE Inc.)	E6AP	+
	KIAA0317	AREL1; FIEL1	-
	UBE3B (Sino Biological Inc.)	KOS; BPIDS	+++
	UBE3C (Sino Biological Inc.)	HECTH2	-
	HUWE1 (Sino Biological Inc.)	MULE; Ib772; LASU1; UREB1; HECTH9; URE-B1; ARF-BP1; HSPC272	++
	TRIP12 (Kelei Shanghai Inc.)	ULF; MRD49; TRIP-12	-
	HACE1 (Sino Biological Inc.)	SPPRS	++
RBR E3s	Parkin (Kelei Shanghai Inc.)		-
