# Supplementary material for: TRIB3 promotes MYC-associated lymphoma development through suppression of UBE3B-mediated MYC degradation
Source: Nat Commun. 2020 Dec 9;11:6316. doi: 10.1038/s41467-020-20107-1 (PMC7725785; doi:10.1038/s41467-020-20107-1)
Supplement: Supplementary file 3 — Reporting Summary [file 41467_2020_20107_MOESM3_ESM.pdf]

nature research

Corresponding author(s): Zhuo-wei Hu

Last updated by author(s): Oct 11, 2020

Reporting Summary

Nature Research wishes to improve the reproducibility of the work that we publish. This form provides structure for consistency and transparency in reporting. For further information on Nature Research policies, see Authors & Referees and the Editorial Policy Checklist.

Statistics

For all statistical analyses, confirm that the following items are present in the figure legend, table legend, main text, or Methods section.

n/a	Confirmed	
☐	☒	The exact sample size (*n*) for each experimental group/condition, given as a discrete number and unit of measurement
☐	☒	A statement on whether measurements were taken from distinct samples or whether the same sample was measured repeatedly
☐	☒	The statistical test(s) used AND whether they are one- or two-sided<br>*Only common tests should be described solely by name; describe more complex techniques in the Methods section.*
☒	☐	A description of all covariates tested
☐	☒	A description of any assumptions or corrections, such as tests of normality and adjustment for multiple comparisons
☐	☒	A full description of the statistical parameters including central tendency (e.g. means) or other basic estimates (e.g. regression coefficient) AND variation (e.g. standard deviation) or associated estimates of uncertainty (e.g. confidence intervals)
☐	☒	For null hypothesis testing, the test statistic (e.g. *F*, *t*, *r*) with confidence intervals, effect sizes, degrees of freedom and *P* value noted<br>*Give P values as exact values whenever suitable.*
☒	☐	For Bayesian analysis, information on the choice of priors and Markov chain Monte Carlo settings
☒	☐	For hierarchical and complex designs, identification of the appropriate level for tests and full reporting of outcomes
☐	☒	Estimates of effect sizes (e.g. Cohen's *d*, Pearson's *r*), indicating how they were calculated

Our web collection on statistics for biologists contains articles on many of the points above.

Software and code

Policy information about availability of computer code

Data collection	Standard software and the respective analysis tools provided by manufacturers were listed in the methods (Olympus microsystems, Bioer LineGene 9620 PCR, GE BIAcore T200, etc.). No software was used other than that listed in the Methods.
Data analysis	Student's t-test, Pearson's correlation test and Kaplan-Meier analysis were done by using Prism GraphPad 7.0.<br>GSEA analysis was conducted using MSigDB v6.1. Quantitative image analysis was performed with Imaris 9.3.1. The dissociation constant (KD) was calculated according to the BIA-evaluation software. All flow cytometry data were analyzed using FCS Express 6.<br>Immunohistochemistry analysis was performed by Image-Pro Plus 5.1. Western blots images were analyzed by Gel Pro Analyzer 3.2.

For manuscripts utilizing custom algorithms or software that are central to the research but not yet described in published literature, software must be made available to editors/reviewers. We strongly encourage code deposition in a community repository (e.g. GitHub). See the Nature Research guidelines for submitting code & software for further information.

Data

Policy information about availability of data

All manuscripts must include a data availability statement. This statement should provide the following information, where applicable:

- Accession codes, unique identifiers, or web links for publicly available datasets
- A list of figures that have associated raw data
- A description of any restrictions on data availability

The RNA-seq and ChIP-seq data have been uploaded to the NCBI Gene Expression Omnibus (GEO) database under accession numbers GSE117128, GSE126258, GSE143862, GSE143863, GSE143864, GSE143865 and GSE143866, respectively. The TRIB3 mRNA expression was analyzed on the following accession code GSE6338, GSE24881 and the web site http://llmpp.nih.gov/DLBCL/. All other data supporting the findings of this study are available from the corresponding author upon reasonable request. A Reporting Summary for this study is available as a Supplementary Information file. The uncropped blot figures, and original data underlying Figs. 1–8 and Supplementary Figs. 1–8 are provided as a Source Data file. Source data are provided with this paper.

Field-specific reporting

Please select the one below that is the best fit for your research. If you are not sure, read the appropriate sections before making your selection.

☒ Life sciences ☐ Behavioural & social sciences ☐ Ecological, evolutionary & environmental sciences

For a reference copy of the document with all sections, see nature.com/documents/nr-reporting-summary-flat.pdf

Life sciences study design

All studies must disclose on these points even when the disclosure is negative.

Sample size	For in vitro experiments, at least three biological replicates were achieved for most of the experiments, except for the chip-seq. Such sample sizes are typical for the in vitro experiments and sufficient for a statistical analysis. For in vivo experiments, a sample size of n = 6-14 mice was used per experimental group. Sample size was determined based on our previous experience (Nat Commun. 2015;6:7951; Cancer Cell. 2017;31(5):697#710; Gastroenterology. 2019;156(3):708#721), which is sufficient to generate statistically significant results. No statistical method was used to predetermine sample size.
Data exclusions	No data were excluded from the analysis.
Replication	Most of the in vitro experiments were repeated independently at least three times except for those specifically indicated in the figure legends. Multiple mice (n = 6-14/group) were used for every in vivo experiment. All the attempts at replication were successful.
Randomization	For animal studies, the mice were earmarked before grouping and then were randomly separated into groups by an independent person; however, no particular method of randomization was used.or other experiments, cells/samples were randomly assigned to groups to avoid bias.
Blinding	Experimenters were blinded to group allocation for IHC staining and grading, quantification of CFU numbers. All other experiments were performed in a non-blinded manner, because the experimental design was complicated, the researchers were limited, and blinding feasibility was poor.

Reporting for specific materials, systems and methods

We require information from authors about some types of materials, experimental systems and methods used in many studies. Here, indicate whether each material, system or method listed is relevant to your study. If you are not sure if a list item applies to your research, read the appropriate section before selecting a response.

Materials & experimental systems

n/a	Involved in the study	
☐	☒	Antibodies
☐	☒	Eukaryotic cell lines
☒	☐	Palaeontology
☐	☒	Animals and other organisms
☐	☒	Human research participants
☒	☐	Clinical data

Methods

n/a	Involved in the study	
☐	☒	ChIP-seq
☐	☒	Flow cytometry
☒	☐	MRI-based neuroimaging

Antibodies

Antibodies used	Western Blots: anti-TRIB3 (Abcam, ab75846, 1:1000), anti-TRIB3 (ThermoFisher, PA5-15480, 1:1000), Anti-TRIB3(Abcam, ab137526, 1:1000), TRB-3 Antibody (D-4) (Santa cruz Biotechnology, sc-365842, 1:500), anti-GAPDH (ZSGB-BIO TA-08, 1:2000) anti-MYC (D3N8F) (CST, #13987S, 1:1 000), anti-MYC Antibody (CST, #9402S, 1:1000), anti-UBE3B (Abcam, ab83834,1:1000), anti-β-Actin (D6A8) (CST,# 8457S,1:1000), anti-Ubiquitin (CST, #3933S, 1:1000), anti-Fbxw7 (Abcam,ab109617,1:1000), anti-Max (S20) (CST, #4739,1:1000), anti- HUWE1 (Abnova, PAB12996, 1:1000), anti-Phospho-c-Myc (Thr58) (E4Z2K) (CST, #46650,1:1000), anti-Phospho-c-Myc (Ser62) (E1J4K) (CST, #13748,1:1000), anti-UBE3A [EPR7330](Abcam, ab126765,1:1000),anti-UBE3C (Abcam, ab226173,1:1000),anti-TRIB2 (Abcam, ab117981,1:1000), anti-TRIB1 (Abcam, ab137717,1:1000), anti-TRIM32 (Abcam, ab131223, 1:1000), anti-Myc (MBL, #562, 1:1000), anti-Myc (MBL, M047-3, 1:1000), anti-GFP (MBL, #598, 1:1000), anti-GFP (MBL, M048-3, 1:1000), anti-DDDDK (MBL, PM020,1:1000), anti-DDDDK (MBL, M185-3L,1:1000),anti-HA (MBL, 561), anti-HA (MBL, M180-3,1:1000), anti-His (MBL, PM032,1:1000), anti-His (MBL, D291-3,1:1000). Immunofluorescence& Immunohistochemistry: anti-TRIB3 (Abcam, ab137526, 1:100), anti-MYC (R&D, AF3696, 1:100), anti-MYC (R&D, NB600-302, 1:100), anti-Ki67 [SP6] (Abcam Ab16667,1:100), anti-UBE3B (Invitrogen, PA5-59390, 1:100), anti-TRIB3 (1H2) (Novus, H00057761-M03,1:100), anti-Max (S20) (CST, #4739,1:100), Alexa Fluor 488 (Thermo Fisher, R37114, 1:200), Alexa Fluor 488 (Thermo Fisher, R37118, 1:200), Alexa Fluor 555 (Thermo Fisher, A-31572,1:200), Alexa Fluor 555 (Thermo Fisher, A-31570,1:200), Alexa Fluor 647 (Thermo Fisher, A-31571, 1:200), Alexa Fluor 647 (Thermo Fisher, A-31573, 1:200). Chip-seq: anti-Rpb1 CTD (4H8) (CST,#2629, 10 ug/ChIP), anti-MYC (D3N8F) (CST, #13987S, 10ug/ChIP). Flow cytometry: FITC anti-human CD19 antibody (Biolegend, 392508, 1:100), PE anti-human CD3 antibody (Biolegend, 981004, 1:100), APC anti-

mouse CD3 antibody (Biolegend, 100236, 1:100), FITC anti-mouse/human CD45R/B220 antibody (Biolegend, 103205,1:100), FITC anti-mouse Ki-67 antibody (Biolegend, 652410, 1:100), PE anti-mouse/human CD11b antibody (Biolegend, 101207, 1:100), FITC anti-human CD11b antibody (Biolegend, 301330,1:100).

Validation

anti-TRIB3, human,WB and IP, (https://www.abcam.com/trib3-antibody-epr3151y-ab75846.html); anti-TRIB3, human and mouse, WB, (https://www.thermofisher.com/cn/zh/antibody/product/TRIB3-Antibody-Polyclonal/PA5-15480); anti-TRIB3, human,WB and IP, (https://www.abcam.cn/trib3-antibody-ab137526.html); anti-TRB-3, human and mouse, WB and IP,(https://www.scbt.com/p/trb-3-antibody-d-4); anti-GAPDH, human and mouse, WB, (http://www.zsbio.com/product/TA-08); anti-c-Myc (D3N8F); anti-c-Myc, human and mouse, WB, IP, IF and ChIP, (https://www.cellsignal.cn/products/primary-antibodies/c-myc-n-myc-d3n8f-rabbit-mab/13987); anti-c-Myc,human and mouse, WB, IP and ChIP, (https://www.cellsignal.cn/products/primary-antibodies/c-myc-antibody/9402?N=4294956287&Ntt=9402s&fromPage=plp); anti-UBE3B, human, WB, (https://www.abcam.cn/ube3b-antibody-ab83834.html); anti-β-Actin (D6A8), human and mouse, WB, (https://www.cellsignal.cn/products/primary-antibodies/b-actin-d6a8-rabbit-mab/8457?N=4294956287&Ntt=8457s&fromPage=plp); anti-Ubiquitin, All species, WB, (https://www.cellsignal.cn/products/primary-antibodies/ubiquitin-antibody/3933 N=4294956287&Ntt=3933s&fromPage=plp); anti-Fbxw7, human, WB and IP, (https://www.abcam.cn/fbxw7-antibody-ab109617.html); anti-Max, human and mouse, WB, IP and IF, (https://www.cellsignal.cn/products/primary-antibodies/max-s20-antibody/4739?N=4294956287&Ntt=4739&fromPage=plp); anti-Huwe1, human and mouse, WB, (http://www.abnova.com/products/products_detail.asp?catalog_id=PAB12996); anti-Phospho-c-Myc (Thr58) (E4Z2K), human and mouse, WB, (https://www.cellsignal.cn/products/primary-antibodies/phospho-c-myc-thr58-e4z2k-rabbit-mab/46650?N=4294956287&Ntt=46650&fromPage=plp); anti-Phospho-c-Myc (Ser62) (E1J4K), human and mouse, WB, (https://www.cellsignal.cn/products/primary-antibodies/phospho-c-myc-ser62-e1j4k-rabbit-mab/13748?N=4294956287&Ntt=13748&fromPage=plp); anti-UBE3A [EPR7330], human and mouse, WB, (https://www.abcam.cn/ube3a-antibody-epr7330-ab126765.html); anti-UBE3C, human, WB and IP, (https://www.abcam.cn/ube3c-antibody-ab226173.html); anti-TRIB2, human, WB, (https://www.abcam.cn/trib2-antibody-oti8d11-ab117981.html); anti-TRIB1, human, WB, (https://www.abcam.cn/trib1-antibody-ab137717.html); anti-TRIM32, human and mouse, WB, (https://www.abcam.com/trim32-antibody-ep6355-ab131223.html); anti-Myc, human and mouse, WB and IP, (https://ruo.mbl.co.jp/bio/e/dtl/A/index.html?pcd=562); anti-Myc, human and mouse, WB and IP, (https://ruo.mbl.co.jp/bio/e/dtl/A/?pcd=M047-3); anti-GFP, human and mouse, WB and IP, (https://ruo.mbl.co.jp/bio/e/dtl/A/?pcd=598); anti-GFP, human and mouse, WB and IP, (https://ruo.mbl.co.jp/bio/e/dtl/A/?pcd=M048-3); anti-DDDDK, human and mouse, WB and IP, (https://ruo.mbl.co.jp/bio/e/dtl/A/?pcd=PM020); anti-DDDDK, human and mouse, WB and IP, (https://ruo.mbl.co.jp/bio/e/dtl/A/?pcd=M185-3L); anti-HA, human and mouse, WB and IP, (https://ruo.mbl.co.jp/bio/e/dtl/A/?pcd=561); anti-HA, human and mouse, WB and IP, (https://ruo.mbl.co.jp/bio/e/dtl/A/?pcd=M180-3); anti-His, human and mouse, WB and IP, (https://ruo.mbl.co.jp/bio/e/dtl/A/?pcd=PM032); anti-His, human and mouse, WB and IP, (https://ruo.mbl.co.jp/bio/e/dtl/A/?pcd=D291-3); Immunofluorescence& anti-TRIB3, human, WB,IHC, ICC/IF, (https://www.abcam.cn/trib3-antibody-ab137526.html); anti-c-Myc, human and mouse, WB, IP, IHC,ICC/IF, (https://www.novusbio.com/products/c-myc-antibody_af3696); anti-c-Myc, human and mouse, WB, IP, IHC,ICC/IF, (https://www.novusbio.com/products/c-myc-antibody-9e10_nb600-302); anti-Ki67 [SP6], human and mouse, WB, IHC,ICC/IF, (https://www.abcam.com/ki67-antibody-sp6-ab16667.html); anti-UBE3B, human, IHC,ICC/IF,(https://www.thermofisher.com/antibody/product/UBE3B-Antibody-Polyclonal/PA5-59390); anti-TRIB3 (1H2), human, WB, ICC/IF, (novusbio.com/products/trib3-antibody-1h2_h00057761-m03); Alexa Fluor 488, all, IF, (https://www.thermofisher.com/antibody/product/Donkey-anti-Mouse-IgG-H-L-Secondary-Antibody-Polyclonal/R37114); Alexa Fluor 488, all, IF, (https://www.thermofisher.com/antibody/product/Donkey-anti-Rabbit-IgG-H-L-Secondary-Antibody-Polyclonal/R37118); Alexa Fluor 555,all, IF, (https://www.thermofisher.com/antibody/product/Donkey-anti-Rabbit-IgG-H-L-Highly-Cross-Adsorbed-Secondary-Antibody-Polyclonal/A-31572); Alexa Fluor 555,all, IF, (https://www.thermofisher.com/antibody/product/Donkey-anti-Mouse-IgG-H-L-Highly-Cross-Adsorbed-Secondary-Antibody-Polyclonal/A-31570); Alexa Fluor 647, all, IF,(https://www.thermofisher.com/antibody/product/Donkey-anti-Mouse-IgG-H-L-Highly-Cross-Adsorbed-Secondary-Antibody-Polyclonal/A-31571); Alexa Fluor 647, all, IF, (https://www.thermofisher.com/antibody/product/Donkey-anti-Rabbit-IgG-H-L-Highly-Cross-Adsorbed-Secondary-Antibody-Polyclonal/A-31573); Chip-seq: anti-Phospho-Rpb1 CTD (Ser5) (D9N5I), human and mouse, WB,IP and Chip,(https://www.cellsignal.com/products/primary-antibodies/phospho-rpb1-ctd-ser5-d9n5i-rabbit-mab/13523?Ntk=Products&Ntt=13523); anti-Phospho-Rpb1 CTD (Ser2) (E1Z3G) , human and mouse, WB,IP and Chip, (https://www.cellsignal.com/products/primary-antibodies/phospho-rpb1-ctd-ser2-e1z3g-rabbit-mab/13499?Ntk=Products&site-search-type=Products&N=4294956287&Ntt=anti-phospho-rpb1+ctd+%2528ser2%2529+%2528e1z3g%2529&fromPage=plp); anti-Rpb1 CTD (4H8), human and mouse, WB,IP and Chip, (https://www.cellsignal.com/products/primary-antibodies/rpb1-ctd-4h8-mouse-mab/2629?Ntk=Products&_=1602303087465&Ntt=anti-rpb1%2520ctd%2520(4h8)&tahead=true); anti-MYC (D3N8F), human and mouse, WB,IP and Chip, (https://www.cellsignal.com/products/primary-antibodies/c-myc-n-myc-d3n8f-rabbit-mab/13987?Ntk=Products&site-search-type=Products&N=4294956287&Ntt=anti-c-myc+%2528d3n8f%2529+&fromPage=plp); Flow cytometry: FITC anti-human CD19 antibody, human, IF, (https://www.biolegend.com/en-us/products/fitc-anti-human-cd19-antibody-16221); PE anti-human CD3 antibody, human, IF,(https://www.biolegend.com/en-us/products/pe-anti-human-cd3-antibody-13257); APC anti-mouse CD3 antibody, mouse, IF, (https://www.biolegend.com/en-us/products/apc-anti-mouse-cd3-antibody-8055); FITC anti-mouse/human CD45R/B220 antibody, human and mouse, IF, (https://www.biolegend.com/en-us/products/fitc-anti-mouse-human-cd45r-b220-antibody-445), FITC anti-mouse Ki-67 antibody, mouse, IF, (https://www.biolegend.com/en-us/products/fitc-anti-mouse-ki-67-antibody-8573), PE anti-mouse/human CD11b antibody, human and mouse, IF, (https://www.biolegend.com/en-us/products/pe-anti-mouse-human-cd11b-antibody-349), FITC anti-human CD11b antibody, human, IF, (https://www.biolegend.com/en-us/products/fitc-anti-human-cd11b-antibody-8299).

Eukaryotic cell lines

Policy information about cell lines

Cell line source(s)

H9 (T cell lymphoma) cells, NAMALWA (BL) cells, K562 (chronic myelogenous leukemia) cells, MOLT-4 (acute lymphoblastic leukemia) cells, bjab (BL cells), Jurkat (acute T cell leukemia) cells, Sup-B15 (acute B cell leukemia) cells, U937 (myeloid leukemia) cells, NALM6 (acute B cell leukemia) cells, BV173 (chronic myeloid leukemia) cells, Raji (BL) and HEK293T cells were purchased from Shanghai Bioleaf Biotech Co.,Ltd.

Authentication	All the cell lines were recently authenticated by STR profiling.
Mycoplasma contamination	Cell lines were routinely tested for potential mycoplasma contamination by using commercial mycoplasma detection kits (Lonza, LT07-418). All tests were negative.
Commonly misidentified lines (See ICLAC register)	No commonly misidentified cell lines were used.

Animals and other organisms

Policy information about studies involving animals; ARRIVE guidelines recommended for reporting animal research

Laboratory animals	NNOD-scid IL2Rgnull (NSG) mice (4-6 weeks old, male) were purchased from the Nanjing Biomedical Research Institute of Nanjing University (Nanjing, China). Trib3-knockout (Trib3loxP/loxP, Trib3F/F) mice (5-6 weeks old, male) were generated as described in our previous study (Li et al., 2017). CreERT2 (B6. Cg-Tg(CAG-cre/Esr1*)5Amc/J) mice (5-6 weeks old, 1 male and 2 females) (The Jackson Laboratory, 004682) were obtained from The Jackson Laboratory (CA, USA). When CreERT2 transgenic mice bred with mice containing loxP-flanked sequences, tamoxifen-inducible Cre-mediated recombination results in deletion of the floxed sequences in widespread cells/tissues of the offspring. CreLck (B6. Cg-Tg(Lck-cre)548Jxm/J) mice (5-6 weeks old, 1 male and 2 females) (The Jackson Laboratory, 003802), and CreCD19 (B6.129P2(C)-Cd19tm1(cre)Cgn/J) mice (5-6 weeks old, 1 male and 2 females) (The Jackson Laboratory, 006785) were obtained from the Shanghai Research Center for Model Organisms (Shanghai, China). B6.129P2-Lyz2tm1(cre)/Nju (LysM-Cre) mice(5-6 weeks old, 1 male and 2 females) (N000056) were obtained from Model Animal Resource Information Platform. Myeloid cell-specific, thymocyte-specific, and B lymphocyte-specific Trib3-knockout mice were generated by crossing Trib3F/F mice with CreLysm mice, CreLck mice and CreCD19 mice, respectively. The generation of inducible Trib3-knockout mice was performed by breeding Trib3F/F mice with CreERT2 mice and treating them with tamoxifen to induce Trib3 gene deletion. Lymphomagenesis was induced by transgenic Eu-driven Myc (MycEu). MycEu mice (5-6 weeks old, 1 male and 2 females) were obtained from the Nanjing Biomedical Research Institute of Nanjing University (Nanjing, China, N000116). To determine the role of Trib3 in lymphomagenesis, MycE[illegible] mice were crossed with CreLysmTrib3F/F, CreLckTrib3F/F, CreCD19Trib3F/F, and CreERT2 Trib3F/F mice. For the induced deletion of Trib3 in MycEuCreERT2Trib3F/+ mice, 3-month-old MycEuCreERT2Trib3F/+ mice were treated with tamoxifen (Sigma Aldrich, 06734) at a dose of 250 mg/kg i.p. for 6 days, followed by tamoxifen injection once per week until sacrifice at 6 months. Deletion of Trib3 was corroborated by quantitative PCR and immunoblot analysis. Animals were housed in groups of 4-6 mice per individually ventilated cage in a 12 h light/dark cycle (07:30-19:30 light. 19:30-7:30 dark), with controlled room temperature (23 ± 2°C) and relative humidity (40-50 %).
Wild animals	The study did not involve wild animals.
Field-collected samples	No field-collected samples were used in this study.
Ethics oversight	All animal procedures were conducted in accordance with the guidelines of the Institutional Committee for the Ethics of Animal Care and Treatment in Biomedical Research of Chinese Academy of Medical Sciences and Peking Union Medical College.

Note that full information on the approval of the study protocol must also be provided in the manuscript.

Human research participants

Policy information about studies involving human research participants

Population characteristics	Informed consent was obtained from all participants in accordance with the Declaration of Helsinki. Patient-related information is identified in Supplementary Table 1.
Recruitment	Human lymphoma patient specimens were obtained from Anyang Tumor Hospital, Henan University of Science and Technology and the Institute of Hematology and Blood Diseases Hospital of PUMC.
Ethics oversight	The procedure was approved by the institutional review board at Anyang Tumor Hospital and the Ethics Committee of the Institute of Hematology and Blood Diseases Hospital of PUMC (KT2019055-EC-1).All participants provided written informed consent to publish information that identifies individuals. Our study is compliant with the 'Guidance of the Ministry of Science and Technology (MOST) for the Review and Approval of Human Genetic Resources', which requires formal approval for the export of human genetic material or data from China.

Note that full information on the approval of the study protocol must also be provided in the manuscript.

ChIP-seq

Data deposition

☒ Confirm that both raw and final processed data have been deposited in a public database such as GEO.

☒ Confirm that you have deposited or provided access to graph files (e.g. BED files) for the called peaks.

Data access links<br>*May remain private before publication.*	The Chip-seq data have been uploaded to the GEO database under accession number GSE143863,GSE143864,GSE143865, and GSE143866.
Files in database submission	Ip-Ctrl-Cas9_ChIPSeq, Ip-TRIB3-Cas9_ChIPSeq, Input-Ctrl-Cas9_ChIPSeq, Input-TRIB3-Cas9_ChIPSeq, IP-PCON_ChIPSeq, IP-

Files in database submission	PCM4_ChIPSeq, INPUT-PCON_ChIPSeq, INPUT-PCM4_ChIPSeq, IP-c-Myc(WT)_ChIPSeq, IP-K427R_ChIPSeq, INPUT-c-Myc(WT)_ChIPSeq, INPUT-K427R_ChIPSeq, Ip-Ad-CTRL_ChIPSeq, Ip-Ad-UBE3B_ChIPSeq, Input-Ad-CTRL, Input-Ad-UBE3B.
Genome browser session (e.g. UCSC)	Human genome (Homo_sapiens.GRCh38.87v2)

Methodology

Replicates	There are no biological replicates for ChIP-seq in this study.
Sequencing depth	The total number of reads is 20M, the uniquely mapped reads is around 35 million, the length of reads is 150bp and they are paired-end.
Antibodies	ChIP grade antibodies specific to c-Myc (Cell Signaling Technology, #13987S, 10 ug/ChIP) or RNAPII (Cell Signaling Technology, #2629, 10 ug/ChIP) were coupled to magnetic beads and incubated with the chromatin.
Peak calling parameters	Unambiguously mapped reads were retained for subsequent generation of binding profiles, heatmaps and calling of peaks. MACS2 (Model-based Analysis of ChIP-seq, version 2) was used to identify regions in ChIPed samples of the signal enriched over the background signal from the corresponding input sample, and P value of 10–9 was used as the cutoff to identify statistically significant peaks. Mapped reads were visualized using the Integrative Genomics Viewer (IGV).
Data quality	Reads were quality controlled using FASTQC. Reads with a mapping quality score >30 were retained for analysis. To ensure data quality, we used the Q30, about 500 peaks are at FDR 5% and above 5-fold enrichment.
Software	The total number of reads aligned to the mouse mm9 genome assembly was used as a normalization factor to scale ChIP-seq data sets produced from equal cell numbers. Bamliquidator (https://github.com/BradnerLab/pipeline/wiki bamliquidator, version 1.0) was used to calculate the ChIP-seq read density over a given genomic coordinate.Heatmaps and genome-wide correlation analyses were generated using DeepTools2. To create density distributions around TSS or ChIP peaks and heatmaps indicating c-Myc or RNA polymerase II occupancies, plotHeatmap was used. Peak annotations were achieved using the 'closestBed' feature from the Bedtools suite v2.20.1.

Flow Cytometry

Plots

Confirm that:

☒ The axis labels state the marker and fluorochrome used (e.g. CD4-FITC).

☒ The axis scales are clearly visible. Include numbers along axes only for bottom left plot of group (a 'group' is an analysis of identical markers).

☒ All plots are contour plots with outliers or pseudocolor plots.

☒ A numerical value for number of cells or percentage (with statistics) is provided.

Methodology

Sample preparation	Described in Methods
Instrument	FACSCanto II flow cytometer (BD).
Software	FCS Express 6
Cell population abundance	No sorting was performed
Gating strategy	Gating strategy is provided in the supplementary Figure 2a, 2b, and 6n.

☒ Tick this box to confirm that a figure exemplifying the gating strategy is provided in the Supplementary Information.
